# LacR and TrxB are key virulence factors involved in pneumococcal meningitis as identified by a genome-wide association study

**DOI:** 10.1038/s42003-026-10638-6

**Published:** 2026-07-08

**Authors:** Rutger Koning, Kin Ki Jim, Philip H. C. Kremer, Hsueh-Chien Raymond Cheng, Valery Jaspers, Mans Perez, Bart Ferwerda, Bevika Sewgoolam, Roy Ummels, Maikel Boot, John A. Lees, Stephen D. Bentley, Jan-Willem Veening, Christina M.J.E. Vandenbroucke-Grauls, Matthijs C. Brouwer, Diederik van de Beek

**Affiliations:** 1 Department of Neurology, Amsterdam UMC location University of Amsterdam, Amsterdam Neuroscience, Amsterdam, The Netherlands; 2 Department of Medical Microbiology and Infection Prevention, Amsterdam UMC location VU Universiteit Amsterdam, Amsterdam, The Netherlands; 3 Department of Fundamental Microbiology, Faculty of Biology and Medicine, University of Lausanne, Lausanne, Switzerland; 4 Department of Neurology, Leiden University Medical Center, Leiden University, Leiden, The Netherlands; 5 The Centre for Human Drug Research, Leiden, The Netherlands; 6 European Molecular Biology Laboratory, European Bioinformatics Institute EMBL-EBI, Hinxton, Cambridgeshire UK; 7 The Wellcome Trust Sanger Institute, Wellcome Trust Genome Campus, Hinxton, Cambridge, UK; 8 Department of Clinical Epidemiology and Biostatistics, Amsterdam UMC location University of Amsterdam, Amsterdam, The Netherlands; 9 Department of Clinical Epidemiology, Infectious Diseases Surveillance and Epidemiology, Aarhus University, Aarhus, Denmark

**Keywords:** Meningitis, Bacterial pathogenesis

## Abstract

Pneumococcal meningitis is the most common form of bacterial meningitis, with high case fatality rates. Bacterial genetics influence disease severity, but the underlying mechanisms remain poorly understood. Here, we evaluated top variants from a genome-wide association study (GWAS) linking *Streptococcus pneumoniae* genetic variation to disease outcome. Fifteen *S. pneumoniae* knockout mutants were tested for virulence in a zebrafish embryo meningitis model; six showed reduced virulence compared to wild-type, but four were excluded following competitive index and growth assays. Loss of *SPV_1044 (lacR)* and *SPV_1393 (trxB)* resulted in lower bacterial loads, impaired neuroinvasion and a reduced inflammatory response. Furthermore, deletion of *lacR*, encoding a regulator of galactose metabolism, led to delayed growth in galactose and human cerebrospinal fluid. Deletion of *trxB*, encoding thioredoxin reductase, increased susceptibility to oxidative stress. This proof-of-concept study, combining pathogen GWAS with in vivo validation, identifies two pneumococcal virulence genes linked to disease severity.

## Abstract (web summary)

Pathogen GWAS and in vivo zebrafish validation reveals lactose phosphotransferase system repressor LacR and the thioredoxin reductase TrxB of *Streptococcus pneumoniae* as virulence factors in meningitis.

## Introduction

*Streptococcus pneumoniae* is the leading cause of bacterial meningitis, a severe infection of the membranes surrounding the brain^1^. Despite treatment with antibiotics and adjunctive dexamethasone, mortality and morbidity remain high^2–4^. Mortality rates in pneumococcal meningitis range from 20 to 51%, with higher mortality in low-income countries^3,5^. Among survivors, many suffer from long-term sequelae, including hearing loss and cognitive impairment, whereas others recover without lasting disability^6,7^. Although the reasons for differences in patient outcomes are not fully understood, both host as well as pathogen characteristics are known to play a role^8^. On the pathogen’s side, the capsular serotype and bacterial loads in cerebrospinal fluid have been associated with disease outcomes^9,10^. Understanding the interplay between bacterial genetics and the host response in pneumococcal meningitis could pave the way for novel therapeutic interventions.

We previously showed in a genome-wide association study (GWAS) of human and bacterial genomes from a prospective meningitis cohort that both host and pneumococcal genetic variations contribute significantly to the course of pneumococcal meningitis^11^. On the host side, genetic variation accounted for nearly half of disease susceptibility and approximately one-third of the variation in disease severity. On the pathogen side, pneumococcal genetic variation explained approximately 70% of the pathogen’s invasive potential, but no statistically significant bacterial loci were associated with disease severity. These findings suggest that host factors largely determine the outcome in pneumococcal meningitis, while bacterial factors determine the initial ability to invade the host. Despite the lack of statistically significant bacterial loci associated with disease severity, we hypothesised that genes near suggestive signals or involved in biologically relevant pathways might still influence clinical outcome in patients with pneumococcal meningitis.

In this study, we evaluated the role of these genetic loci identified through the GWAS by constructing pneumococcal gene knockout mutant strains and evaluating their role using an in vivo zebrafish embryo pneumococcal meningitis model. Subsequently, we performed in-depth analyses to elucidate the potential molecular and cellular mechanisms by which these pneumococcal virulence factors contribute to the pathogenesis of pneumococcal meningitis.

## Results

**Fig. 1:**
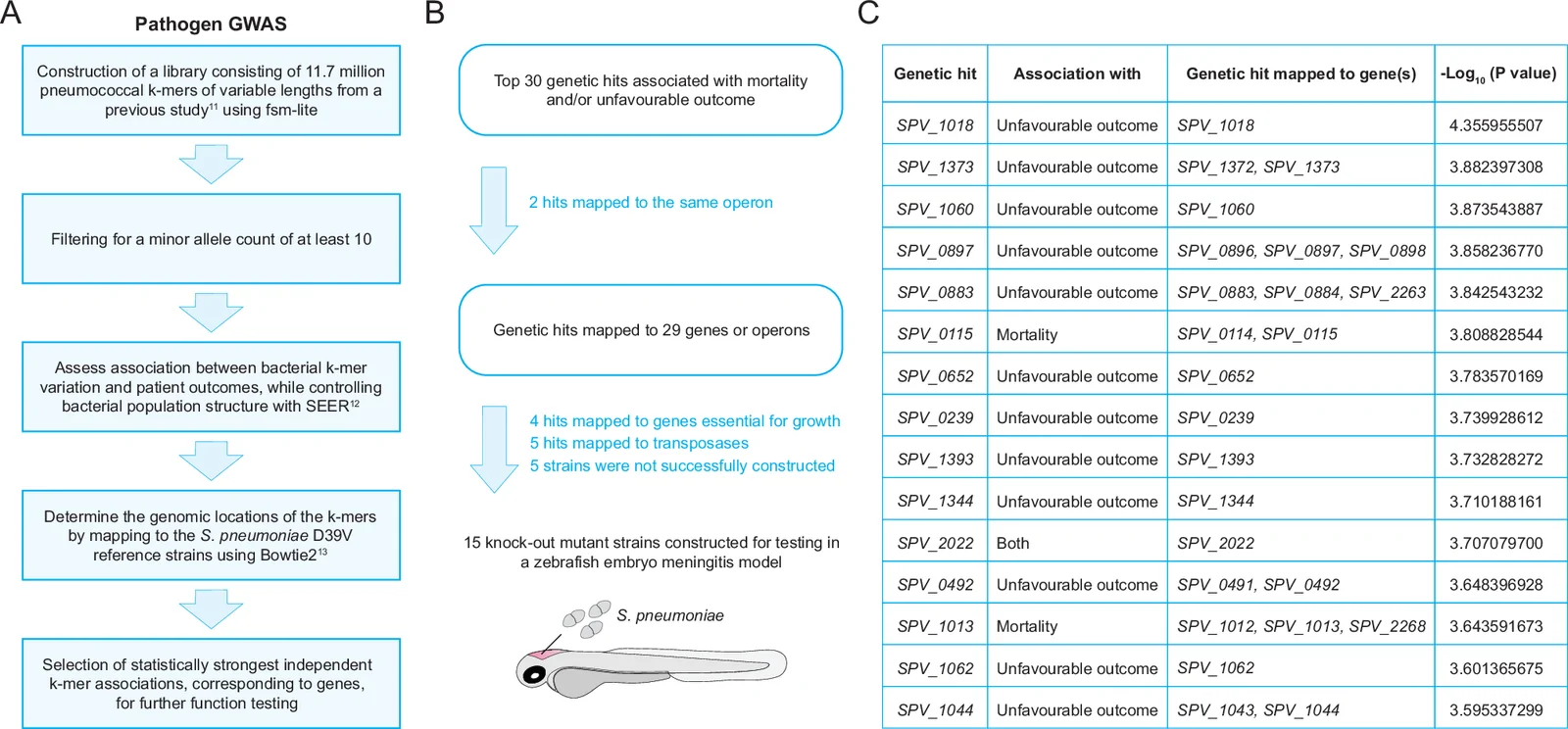
Pathogen GWAS and validation in a zebrafish embryo meningitis model. **A** Schematic illustrating the approach used for genome-wide association studies (GWAS) in *Streptococcus pneumoniae*, linking genetic variants with clinical outcomes in patients with pneumococcal meningitis. **B** Flowchart summarising the strategy for subsequent evaluation of the top 30 pneumococcal genetic loci associated with meningitis severity or patient outcomes evaluation and functional validation using a zebrafish embryo pneumococcal meningitis model. **C** Overview of the 15 genetic hits that were functionally validated. Genetic hits are ranked by significance level (−log₁₀ P value).

A GWAS previously conducted by our group evaluated the association between pneumococcal genetic variation in 1144 isolates from bacterial meningitis patients and clinical outcomes^11^. The presence of unique bacterial sequences (k-mers) was associated with mortality or unfavourable outcome, defined as a Glasgow Outcome Scale of one to four. The k-mers were mapped to genomic regions on the *S. pneumoniae* D39V reference strain (Fig. 1A). After correcting for multiple testing, no genomic region was associated with mortality or unfavourable outcome at the genome-wide significance level with a stringent cut-off (*P* < 5.0 × 10^−8^). However, true signals might fall below this statistical threshold, due to sample size constraints and the small effect size of individual hits for complex traits such as clinical outcome. We therefore further examined the most strongly associated genetic hits to explore biological relevance and generate hypotheses for future studies. We ranked the genetic hits associated with mortality or unfavourable outcome by significance and evaluated the top 30, which had P values from 3.69 × 10^−5^ to 2.56 × 10^−4^ (Fig. 1B, C, Supplementary Table 1). To functionally assess the associated genomic regions, knockout mutant strains targeting the gene(s) within these loci were constructed. Hits that mapped to intergenic regions, transposases or genes known to be essential for growth in vitro were not further tested (Supplementary Table 1). A total of 15 knockout mutant strains were constructed (Fig. 1C).

### The zebrafish embryo meningitis model identifies multiple genes with decreased virulence

**Fig. 2:**
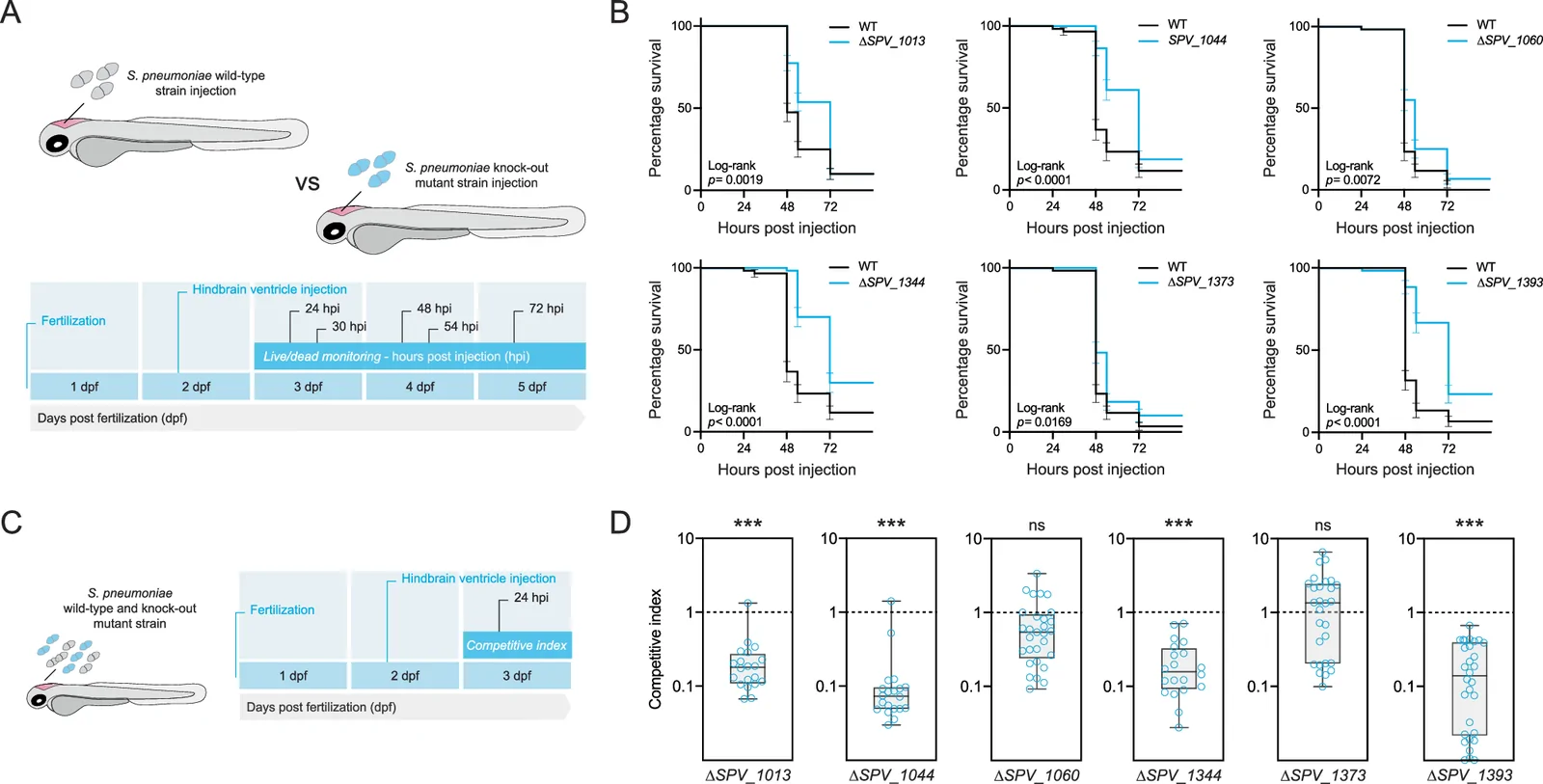
Identification of new pneumococcal virulence genes using pathogen genomics and validation in the zebrafish embryo meningitis model. **A** Experimental set-up and timeline of survival experiments in the zebrafish embryo meningitis model. **B** Survival curves of zebrafish embryos infected in the hindbrain ventricle with ~300 colony forming units (CFU) (range 210–400) wild-type (black) or knockout (blue) strains. Zebrafish were infected by hindbrain ventricle injection and followed for survival for 72 h. The data represent the mean ± SEM of three biological replicates with 20 embryos per group (*n* = 60 embryos in total/group); *P* values were determined by log-rank test. **C** Experimental set-up and timeline of competitive index experiments in the zebrafish embryo meningitis model. **D** Competitive index of knockout mutants. In each competitive index experiment, the wild-type and corresponding knockout strain were injected into the zebrafish hindbrain ventricle at similar inocula, ranging from approximately 90 to 330 CFU per strain across experiments. At 24 hours post-injection (hpi), zebrafish embryos were homogenised, and CFUs were determined. The competitive index was calculated as the ratio of knockout to wild-type strain CFUs at 24 h, normalised to their initial ratio at the time of injection. A competitive index lower than 1 indicates a decreased relative growth of the mutant strain. Each circle represents an individual zebrafish, the bars represent the mean with the interquartile range (25th to 75th percentile) and the whiskers extend to the minimum and maximum values of two biological replicates with 10–15 embryos per group (*n* = 20–30 embryos in total/group). Source data available in Supplementary Data 1. ns indicates not significant, * indicates *P* < 0.05, ** indicates *P* < 0.01, and *** indicates *P* < 0.001; determined by one-sample two-tailed Wilcoxon Signed Rank test t-test.

The possible role of the genes found through GWAS was evaluated in vivo using a zebrafish embryo meningitis model (Fig. 2A). This model is a powerful model for medium throughput screening to identify bacterial mutants with altered virulence^12^. Zebrafish embryos were infected via hindbrain ventricle injection with approximately 300 CFU *S. pneumoniae* D39V wild-type or isogenic knockout mutant strain at 2 days post-fertilisation (dpf) and survival was evaluated for 72 h. Detailed inoculum sizes for all infection experiments are provided in Supplementary Table 2. Infection with 6 of 15 knockout strains significantly increased survival of zebrafish embryos compared to the wild-type strain (Fig. 2B and Supplementary Fig. 1). The largest differences in survival at any time point were observed for *ΔSPV_1013* (76% vs. 48%), *ΔSPV_1044* (86% vs. 37%), *ΔSPV_1060* (55% vs. 23%), *ΔSPV_1344* (98% vs. 37%), *ΔSPV_1373* (48% vs. 23%), and *ΔSPV_1393* (88% vs. 32%). To confirm the reduced virulence of the knockout mutant strains, we also evaluated the relative fitness of the tested knockout mutant strains as compared to the wild-type strain by measuring the competitive index (CI). Both strains were co-injected into the zebrafish hindbrain ventricle to reduce interhost variation at a 1:1 ratio (Fig. 2C). After 24 h, zebrafish were homogenised, and CFUs of each strain were quantified. The competitive index was calculated as the ratio of knockout to the wild-type strain CFUs at 24 h, normalised to their initial ratio at the time of injection. A competitive index of less than one indicates that the wild-type strain outcompetes the knockout strain. Infection with four of the six knockout mutant strains had significantly decreased bacterial outgrowth as compared to the wild-type strain, namely Δ*SPV_1013* (CI 0.18), Δ*SPV_1044* (CI 0.0716), Δ*SPV_1344* (CI 0.17) and Δ*SPV_1393* (CI 0.1420) (Fig. 2D). Next, we evaluated the growth of the mutant strains in a rich chemically defined medium (C + Y medium) in vitro. All knockout mutant strains grew normally in C + Y medium, except for Δ*SPV_1013*, encoding a phage integrase family protein with integrase/recombinase activity, and Δ*SPV_1344*, encoding a hypothetical protein (Supplementary Fig. 2). In summary, we identified two genes, Δ*SPV_1044* (*lacR*) and Δ*SPV_1393* (*trxB*), encoding the lactose phosphotransferase system repressor LacR and the thioredoxin reductase TrxB, respectively, whose deletion reduced virulence and impaired relative growth in the zebrafish model of pneumococcal meningitis compared with the wild-type strain, while maintaining normal growth in vitro.

### Loss of LacR and TrxB reduces bacterial burden, neuroinvasion and host inflammatory responses

**Fig. 3:**
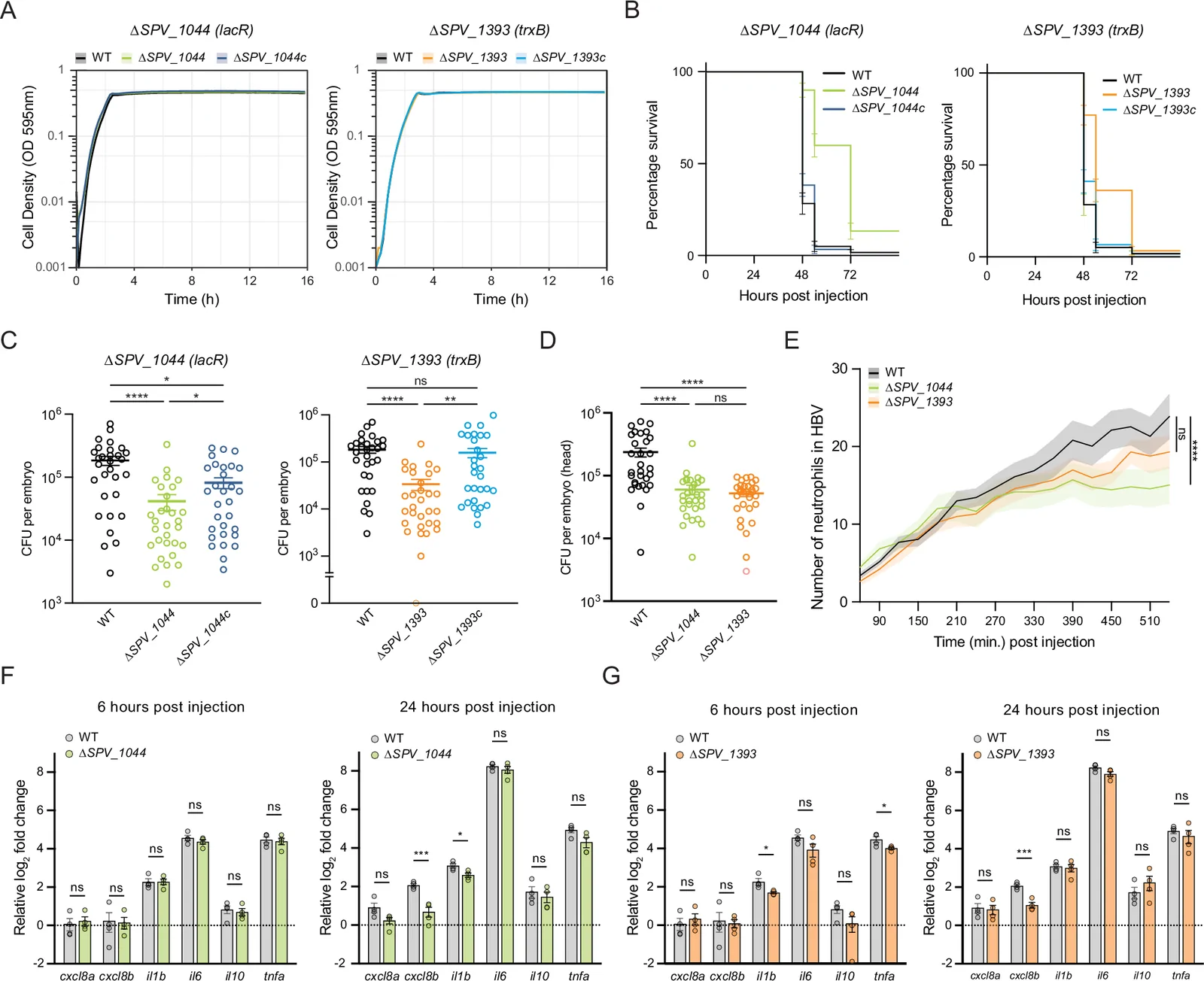
LacR and trxB contribute to pneumococcal virulence, brain invasion, and host inflammatory responses in zebrafish pneumococcal meningitis. **A** Growth curves of *S. pneumoniae* wild-type (WT), the *ΔSPV_1044* (*lacR*) knockout and its complemented strain, and the *ΔSPV_1393* (*trxB*) knockout and its complemented strain in C + Y medium. Data represent the mean ± SD of three technical replicates. **B** Survival curves of 2 days post-fertilisation (dpf) zebrafish embryos injected into the hindbrain ventricle with ~250 CFU (range 210–290) of *S. pneumoniae* D39V: WT (black), *ΔlacR* knockout (green), *ΔlacR* complemented (dark blue), *ΔtrxB* knockout (orange), and *ΔtrxB* complemented (light blue). Data represent the mean ± SEM of three biological replicates with 20 embryos per group (*n* = 60 embryos per group). Statistical analysis was performed using the log-rank test: WT vs. *∆SPV_1044* knockout, *P* < 0.0001; WT vs. *∆SPV1044c*, ns (non-significant); WT vs *∆SPV_1393*, *P* < 0.0001; WT vs *∆SPV_1393c*, ns. **C** Bacterial load in whole zebrafish embryos infected with the indicated strains. Data are from three biological replicates with 10 embryos per group per experiment (*n* = 30 embryos per group). Each circle represents an individual zebrafish; the bar indicates the mean. Statistical analysis was performed using the Kruskal-Wallis test, * = *P* < 0.05, ** = *P* = 0.0040, **** = *P* < 0.0001. **D** Bacterial load in zebrafish embryo head homogenates following bloodstream injection at 2 dpf with the indicated strains at 24 hpi. Data represent the mean ± SEM of three biological replicates with 10 embryos per group (*n* = 30 embryos per group). Each circle represents an individual zebrafish; the bar indicates the mean. Statistical analysis was performed using the Kruskal-Wallis test; **** = *P* < 0.0001, ns non-significant. **E** Neutrophil recruitment to the CNS in zebrafish embryos following hindbrain ventricle injection with ~1000 CFU (range 950–1090) of *S. pneumoniae* WT, the *∆SPV_1044* knockout strain, or the *∆trxB* knockout strain over time. Data are from two biological replicates with 8–10 embryos per group per experiment (*n* = 18 embryos for WT, 18 for ∆*lacR*, 19 for ∆*trxB*). Data are presented as mean ± SEM with shaded area indicating the SEM. Statistical analysis was performed using a two-way repeated measures ANOVA with Geisser-Greenhouse correction. For the *∆lacR* strain, a significant effect of time x strain interaction (F(16, 544) = 4.111, *P* < 0.0001) was observed. For the ∆*trxB* strain, no significant effect of time x strain interaction (F(16, 560) = 0.9554, *P* = 0.5050) was observed. Host inflammatory gene expression in zebrafish embryos following hindbrain ventricle injection with WT or the ∆*lacR* (**F**) or ∆*trxB* (**G**) knockout strains at 6 and 24 hpi, as measured by RT-qPCR. *N* = 4 biologically independent replicates. Source data available in Supplementary Data 1. Statistical analysis was performed using the unpaired two-tailed *t* test: * = *P* < 0.05, *** = *P* = 0.0001, ns non-significant.

To confirm that the observed attenuated virulence phenotype is due to the deletion of *lacR* or *trxB*, respectively, and not because of e.g., polar effects, complementation strains (*∆SPV_1044c* and *∆SPV_1393c*) were constructed and tested. Growth curves showed no differences between the knockout mutants, their complemented strains, and the wild-type strain (Fig. 3A). Subsequent survival experiments showed that complementation largely restored the virulence phenotype, indicating that the decrease in virulence in the knockout mutant strains is caused by the deletion of *lacR* and *trxB*, respectively (Fig. 3B). The knockout phenotypes of the *lacR* and *trxB* mutants were further characterised in vivo in the zebrafish meningitis model. Hindbrain ventricle injection with the *lacR* or *trxB* knockout strains resulted in lower bacterial burdens in embryos compared to injection with the wild-type strain at both 6 and 24 hpi (*ΔlacR* 0.7-fold at 6 hpi and 0.13-fold at 24 hpi, and *ΔtrxB* 0.45-fold at 6 hpi and 0.08-fold at 24 hpi vs wild-type; Fig. 3C and Supplementary Fig. 3). Of note, bacterial burdens in embryos infected with the complemented ∆*lacR* strain were comparable to those in the knockout mutant at 6 hpi, while partial restoration was observed at 24 hpi, yet virulence was largely restored in survival experiments. Following bloodstream infection, both mutants showed a reduced capacity to invade the central nervous system, as reflected by lower pneumococcal burdens in zebrafish head homogenates at 24 hpi (*ΔlacR* 0.39-fold and *ΔtrxB* 0.44-fold reduction vs wild-type; Fig. 3D). Neutrophil recruitment to the CNS also showed a reduced trend over time after hindbrain ventricle injection with both mutants, albeit not statistically significant for the ∆*trxB* strain (*ΔlacR* *P* < 0.0001 and *ΔtrxB* *P* = 0.5; Fig. 3E). Host inflammatory gene expression was altered in zebrafish infected with the mutant strains compared to the wild-type strain (Fig. 3F, G). Of the inflammatory genes examined (*cxcl8a*, *cxcl8b*, *il1b*, *il6*, *il10*, and *tnfa*), only *cxcl8b*, *il1b*, and *tnfa* showed significant differences between wild-type and mutant-infected embryos. For the *lacR* mutant, no significant differences were observed at 6 hpi; however, at 24 hpi, both *cxcl8b* and *il1b* were significantly higher in wild-type infected embryos (2.6-fold for *cxcl8b* and 1.4-fold for *il1b*). For the *trxB* mutant, *il1b* and *tnfa* were significantly higher in wild-type infected embryos at 6 hpi (0.7-fold for *il1b* and 0.7-fold for *tnfa)*, whereas at 24 hpi, only *cxcl8b* remained significantly higher compared to the mutant (2.0-fold). Collectively, these findings support a role for LacR and TrxB in both bloodstream-mediated CNS invasion and bacterial persistence within the CNS during meningitis.

### LacR influences virulence through galactose metabolism

**Fig. 4:**
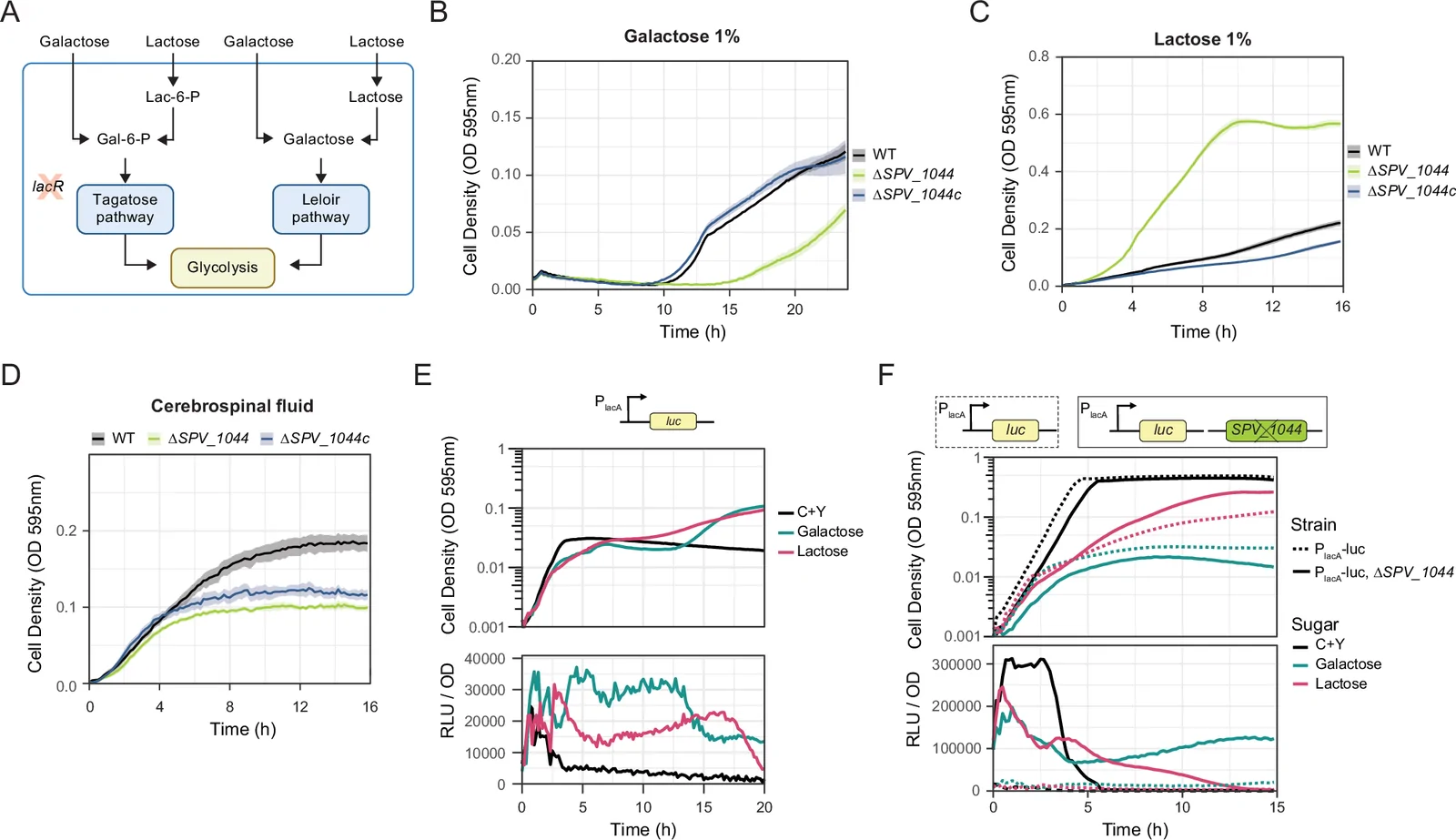
Loss of *LacR* impairs growth on galactose and in human cerebrospinal fluid. Functional evaluation of wild-type (WT), Δ*SPV_1044* knockout mutant (Δ*SPV_1044*) and the complemented (*∆SPV_1044c*) strain. **A** Schematic representation of tagatose and Leloir pathway and the inhibitory role of LacR on galactose metabolism through repression of the tagatose pathway. **B** Growth in C + Y medium supplemented with 1% (w/v) galactose. The lag phase was 10.2 h in the wild-type compared to 14.5 h for the knockout (*P* = 0.028) and 8.2 for the complemented strain (*P* = 0.015). **C** Growth curves in C + Y medium supplemented with 1% (w/v) lactose. The knockout reached a maximum OD_595_ of 0.59 in 10 h with the wild-type reaching OD_595_ 0.14 (*P* < 0.0001) and the complemented strain OD_595_ 0.1 (*P* = 0.02, compared to wild-type) at the same time point. **D** Growth curves in human cerebrospinal fluid. The maximum OD_595_ was 0.2 in the wild-type, compared to OD_595_ 0.12 in the knockout (*P* = 0.005) and OD_595_ 0.14 in the complemented strain (*P* = 0.010). Growth curves were statistically analysed using a two-tailed t-test. OD, optical density. **E** The luciferase reporter gene (*luc*) was placed under the control of the promoter of *lacA* to generate a P*lacA-luc* promoter-fusion strain. Upon addition of 1.25% (w/v) galactose and 0.06% (w/v) lactose to C + Y medium without any sugars, luciferase was highly expressed, indicating these sugars activated the *lacABCD* operon. Luciferase signal is measured by relative luminescence units (RLU) normalised by OD. **F** The P*lacA-luc* promoter-fusion construct was cloned into the Δ*SPV_1044* (*lacR* deletion) background. The deletion of *lacR* leads to increased expression of luciferase and by proxy the expression of the *lacABCD* operon in standard C + Y medium and in C + Y with no sugar, supplemented with 1% lactose and 1% galactose. All growth curve experiments (**B**, **D**–**H**) were performed in three technical replicates and are shown as mean ± SEM. Source data available in Supplementary Data 1. Statistical analysis for growth assays was performed using a two-tailed t-test.

*SPV_1044* (*lacR*) encodes the lactose phosphotransferase system repressor (LacR), which inhibits galactose metabolism through repression of the tagatose pathway (Fig. 4A)^13^. Utilisation of galactose by *S. pneumoniae* has previously been associated with increased virulence^14^. To evaluate the role of *lacR* on sugar metabolism, we performed growth curves in the presence of various sugars that *S. pneumoniae* can utilise as a carbon source. Surprisingly, when grown on galactose the *lacR* mutant had an increased lag phase compared to the wild-type strain (14.5 h vs. 10.2 h; Fig. 4B). In the presence of lactose, loss of *lacR* led to faster growth, with the *lacR* knockout mutant reaching an OD_595_ of 0.59 within 10 h compared to an OD_595_ of 0.14 for the wild-type strain (Fig. 4C). Growth on fructose, GlcNAc, trehalose, mannose, sucrose and maltose was only mildly affected by deletion of *lacR* (Supplementary Fig. 4). These results suggest that uncontrolled processing of galactose is rather toxic to the bacterial cell, likely because of toxic phosphorylated sugar intermediates, and thus needs to be tightly regulated^15^. LacE2F2 is the predicted transporter system for galactose uptake into the tagatose pathway^13^. We hypothesised that deleting *lacE2F2* in the *ΔlacR* background would reduce flux through the pathway and limit toxic sugar-phosphate accumulation. However, loss of *lacE2F2* did not restore the *ΔlacR* mutant’s virulence (Supplementary Fig. 5). Next, we evaluated the growth of the ∆*lacR* mutant in the CSF, which contains relatively high amounts of galactose^16^. Indeed, in CSF, the *lacR* knockout strain showed significantly impaired growth, reaching a maximal density of OD_595_ 0.12 compared to 0.20 for the wild-type strain (Fig. 4D). Growth of the complemented strain in CSF remained partially reduced relative to the wild type (Fig. 4D), despite near complete restoration of the wild-type phenotype in other assays. This incomplete restoration may reflect differences resulting from the chromosomal integration site of the complementation construct, effects that may become apparent under highly restrictive conditions such as CSF. Together, these results suggest that the misregulation of pneumococcal galactose metabolism might lead to decreased virulence of the *lacR* knockout in the CSF. We also constructed a promoter-luciferase fusion to examine gene expression levels of *lacABCD*, a part of the tagatose pathway, in the presence and absence of LacR (Fig. 4E, F). As reported previously, expression of the *lacABCD* operon was activated in the presence of both galactose and lactose and we confirm this (Fig. 4E). Deleting *lacR* led to increased expression of *lacABCD* during the growth phase, which might explain the reduced lag phase in the presence of lactose of the *lacR* mutant compared to wild-type strain (Fig. 4F). Since growth on galactose has previously been linked to changes in the pneumococcal capsule, we evaluated the effect of *lacR* knockout on capsule morphology^13,14^. The capsule was visualised by adding specific antiserum to pneumococcal suspensions, followed by phase contrast microscopy (the Quellung test). No gross changes in capsule morphology were observed between the knockout strains, complemented strains and the wild-type strain after growth on either glucose with sucrose or on galactose (Supplementary Fig. 6). These findings suggest that the reduced virulence of the *lacR*-deficient strain is not attributable to changes in capsule structure.

### TrxB is an essential component for protection against oxidative stress

**Fig. 5:**
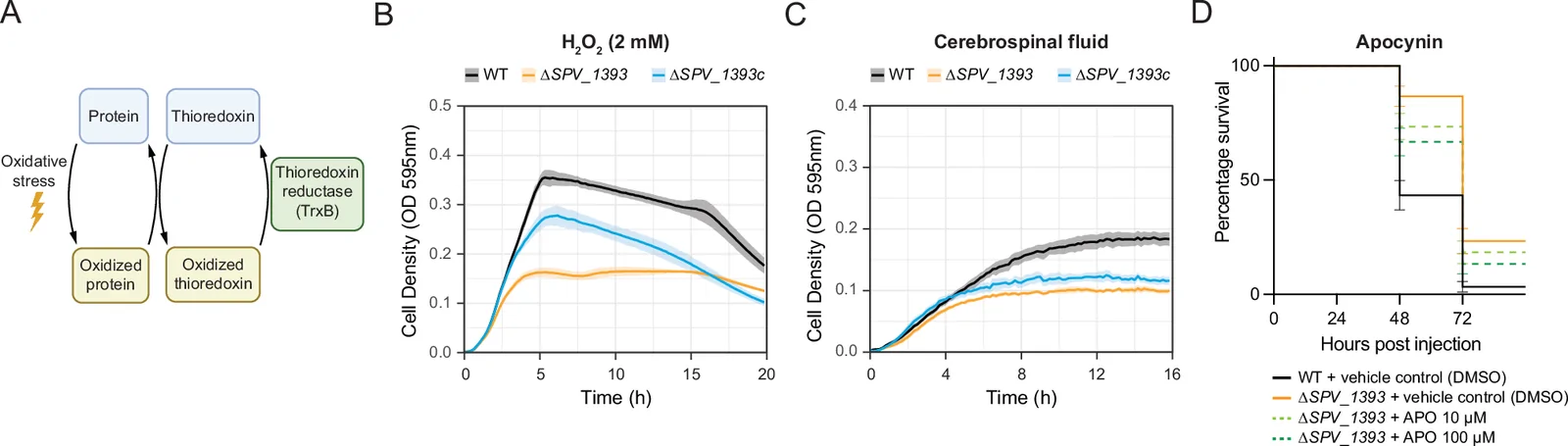
*SPV_1393* (*trxB*) is necessary for protection against radical oxygen species. Functional evaluation of *Streptococcus pneumoniae* wild-type strain (WT, black line), ∆*SPV_1393* (orange line) knockout strain and the ∆*SPV_1393c* complemented strain (blue line). Strains were grown under anaerobic conditions. **A** Schematic representation of the thioredoxin system. **B** Growth curves in C + Y medium supplemented with 2 mM H_2_O_2_. The maximum OD_595_ was 0.38 in the wild-type, compared to 0.20 in the knockout (*P* = 0.002) and 0.30 in the complemented strain (*P* = 0.036). **C** Growth curves in human cerebrospinal fluid. The maximum OD_595_ was 0.20 in the wild-type, compared to 0.12 in the knockout (*P* = 0.006) and 0.14 in the complemented strain (*P* = 0.010). All growth curve experiments (**B**, **C**) were performed in three technical replicates and are shown as mean ± SD. Statistical analysis for growth assays was performed using a two-tailed t-test. **D** Survival analysis of 2 dpf zebrafish embryos injected with *S. pneumoniae* WT (~220 CFU) or ∆*SPV_1393* knockout strain (~260 CFU) into the hindbrain ventricle and treated with 10 or 100 µM apocynin or vehicle control (DMSO). Each experiment represents three biological replicates with 20 embryos per group (*n* = 60 embryos per group). Data represent mean ± SEM. Source data available in Supplementary Data 1. Statistical analysis was performed using the log-rank (Mantel-Cox) test.

*SPV_1393 (trxB)* encodes a thioredoxin reductase, which is an essential part of the antioxidant thioredoxin system (Fig. 5A). Thioredoxins neutralise reactive oxygen species, such as H_2_O_2_, or repair proteins damaged by oxidative stress, for example, by reducing oxidised methionine^17^. In the process, thioredoxins become oxidized and must be reduced by thioredoxin reductase to restore their antioxidant function^17^. The importance of the thioredoxin system in pneumococcal virulence has been demonstrated, but the function of TrxB remains to be elucidated^18^.

The antioxidant role of TrxB was evaluated by growth curves in C + Y medium containing H_2_O_2_. Under oxidative stress, TrxB had a protective effect on growth, as shown in Fig. 5B (maximum growth density of OD_595_ 0.38 for the wild-type strain compared to 0.20 for the knockout mutant strain). In human cerebrospinal fluid (CSF), growth of the knockout mutant strain was also decreased compared to the wild-type strain (OD_595_ 0.12 vs. 0.20) (Fig. 5C). Complementation in these in vitro experiments only partially restored the wild-type phenotype, likely reflecting differences in gene expression or regulation due to the chromosomal integration site of the complementation construct. Finally, to assess whether increased susceptibility to oxidative stress also contributed to the attenuated virulence of the *trxB* mutant in vivo, we repeated the zebrafish survival experiments in the presence of apocynin, a selective inhibitor of NADPH oxidase with antioxidant properties. Inhibition of oxidative stress partially restored the virulence of the *trxB* mutant, supporting a role for *trxB* in resistance to oxidative stress during infection (Fig. 5D).

## Discussion

In this study, we characterised pneumococcal genetic variants most strongly associated with meningitis outcome, as identified in a combined GWAS of patients with pneumococcal meningitis and the infecting strains^11^. Although the initial GWAS did not reveal statistically significant associations between the bacterial genome and disease outcome, this follow-up study provides proof-of-concept that bacterial GWAS results still can be used to identify new pneumococcal virulence factors. Notably, the top hits from the GWAS are genes and alleles conserved in clinical pneumococcal strains from various serotypes, while we performed confirmation assays in a single serotype 2 genetic background (*S. pneumoniae* D39V). Hence, the genes identified in this study are likely to be crucial for meningitis across pneumococcal lineages. This proof-of-concept study suggests that while correcting for multiple testing is essential in bacterial GWAS to prevent false-positive results, it should not be applied too stringently, as this may hinder the identification of novel bacterial virulence genes.

Our findings suggest that loss of *lacR* reduces pneumococcal fitness in vivo by disrupting galactose metabolism regulation. Loss of *lacR* resulted in dysregulation of the tagatose pathway, with marked upregulation of the *lacABCD* operon irrespective of substrate availability. Despite this increased expression, the *lacR* mutant exhibited impaired growth on galactose, as reflected by a prolonged lag phase. One possible explanation is the accumulation of toxic sugar-phosphate intermediates due to unbalanced pathway activity^15^. However, deletion of *lacE2F2*, a phosphotransferase system predicted to mediate galactose uptake into the tagatose pathway, did not restore virulence in vivo. This suggests that alternative mechanisms transport galactose, sustaining metabolic flux through the tagatose pathway. Alternatively, loss of LacR might lead to a fitness defect through the failure to efficiently utilise galactose during infection^19^. Although a detailed mechanistic dissection is beyond the scope of this study, it is likely that tight temporal regulation of carbohydrate utilisation is required during CNS infection. Early in infection, when glucose is relatively available, pneumococci likely preferentially utilise glucose, whereas during later stages when glucose levels decrease in the cerebrospinal fluid, there may be a shift towards alternative carbon sources, including galactose^4,20^. Loss of LacR may disrupt this adaptive metabolic switching, thereby reducing pneumococcal fitness and virulence in vivo^20^.

Consistent with our findings, galactose utilisation has been associated with increased pneumococcal virulence in other models, as mice infected intranasally with pneumococci pre-grown on galactose developed more severe disease than those infected with glucose-grown bacteria^14^. This is likely because in the respiratory tract glucose availability is low and pneumococci use galactose as an alternative carbon source, making galactose metabolism especially important in this setting. Importantly, pneumococci pre-grown on galactose did not have increased virulence in bloodstream infections, where abundant glucose is used as the preferred sugar^19,21^.In the setting of bacterial meningitis, pneumococci are often commensals in the respiratory tract before they cause invasive disease^22^. Growth in the respiratory tract may metabolically adapt pneumococci to metabolise galactose, which could be an advantage in the low glucose environment of the inflammatory CSF^14,16^. Interestingly, preclinical work has shown that targeting galactose metabolism with galactose analogues can suppress pneumococcal growth, indicating its potential value as an antimicrobial drug target^14^.

Furthermore, galactose utilisation has been linked to increased virulence via enhanced capsule synthesis, a critical determinant of pneumococcal resistance to host immune defenses^14^. In pneumococci, galactose catabolism through the Leloir pathway supplies nucleotide sugar precursors essential for capsule production^14^. Our results show no significant changes in capsule morphology between the *lacR* knockout, complement, and wild-type strains after growth on glucose and sucrose, or galactose (Supplementary Fig. 6). This suggests that the reduced virulence of the lacR-deficient strain is not due to changes in capsule production, although we cannot rule out the possibility that *lacR* deficiency influenced capsule composition, thereby reducing virulence.

We showed that TrxB, encoding thioredoxin reductase, contributes to virulence in experimental pneumococcal meningitis. Although the thioredoxin system has been studied in this context, the specific role of TrxB has not been investigated, despite its identification as a pneumococcal virulence factor in two genetic screens in experimental models^23,24^. In experimental pneumococcal meningitis, the thioredoxin system contributes to bacterial virulence by enabling the repair of proteins damaged by oxidative stress^18^. TrxB reduces oxidized thioredoxin, restoring it to the active form and sustaining the antioxidant response. Reactive oxygen species (ROS) are abundant during pneumococcal infection, arising from both the host immune response and bacterial metabolism^24,25^. Consistent with this, inhibition of ROS production with apocynin partially restored the virulence of the *trxB* mutant in vivo, indicating that TrxB is required for pneumococcal resistance to oxidative stress. In addition, the *trxB* knockout strain showed impaired growth in cerebrospinal fluid, potentially reflecting exposure to oxidative stress–inducing components such as complement or antimicrobial peptides^26^. While ROS are essential for bacterial killing during meningitis, they also contribute to host tissue damage^22^. Targeting the thioredoxin system might decrease the levels of ROS necessary to kill *S. pneumoniae*, thereby reducing host tissue damage and making it an attractive target for new antimicrobials.

Our study has limitations. While our k-mer-based bacterial GWAS identified several novel loci associated with susceptibility, none were significantly associated with clinical outcome^11^. Even after statistical correction, determining which k-mers are truly biologically relevant remains challenging. Using k-mers has the advantage of identifying accessory genes that might not be carried in a reference sequence. However, unlike SNPs, which map directly to known genomic positions, k-mers may need further annotation and gene mapping to establish their biological meaning. To investigate candidate genes, we generated knockout strains, creating extreme phenotypes for experimental analysis. However, the k-mers identified may lead to more subtle genomic variation, including small sequence changes, insertions/deletions and copy number variations. As a result, many significant k-mers likely subtly modify protein sequence or gene regulation rather than complete loss-of-function events. Such partial functional variation can fine-tune enzymatic activity, regulatory interactions, metabolic flux, or stress responses in ways that affect bacterial fitness during infection without producing overt phenotypes in gene deletion mutants. Additionally, our experiments were conducted using the *S. pneumoniae* serotype 2 D39V strain, and findings were not validated in other pathogenic strains. However, since the *lacR* and *trxB* genes are both conserved in the core genome, their function is likely conserved across different pathogenic pneumococcal strains.

In conclusion, our study, which integrates pathogen GWAS in patients with pneumococcal meningitis and an experimental zebrafish embryo meningitis model, identified *lacR* and *trxB* as two novel pneumococcal virulence factors linked to disease severity. We confirmed the role of these genes in galactose metabolism and protection against oxidative stress, respectively, highlighting these pathways as potential targets for vaccine development and antimicrobial therapy.

## Methods

### Association of bacterial variation

Previous findings from a k-mer GWAS examining the genetic variation in *S. pneumoniae* associated with patient outcomes in meningitis were utilised to select genomic regions for further functional investigation^11,27,28^. Briefly, a library consisting of 11.7 million pneumococcal k-mers of variable lengths was constructed using fsm-lite, followed by filtering for a minor allele count of at least ten (Fig. 1A)^29^. The association between bacterial k-mer variation and patient outcomes, while controlling for bacterial population structure, was assessed using SEER^29^. Outcome was assessed using the Glasgow Outcome Scale (GOS) score, ranging from a score of 1 = death to a score of 5 = mild or no disability. A favourable outcome was defined as a GOS score of 5, and an unfavourable outcome was defined as a GOS score of 1–4^30^. The genomic locations of the k-mers were determined by mapping them to the *S. pneumoniae* D39V reference strain using Bowtie2 (version 2.4.5)^31^. Finally, the 30 statistically significant independent k-mer associations corresponding to genes were identified, and 15 genes of these genes or operons were functionally validated (Fig. 1B, C and Supplementary Table 1).

### Bacterial strains and growth conditions

The *S. pneumoniae* serotype 2 D39V wild-type strain was used for all experiments and the construction of isogenic mutant strains^31^. All used strains can be found in Supplementary Table 3 and all used oligonucleotides in Supplementary Table 4. Pneumococcal strains were grown overnight on Columbia blood agar plates supplemented with 5% sheep blood (COS) at 37 °C with 5% CO_2_. Subsequently, injection stocks were prepared by suspending bacteria in C + Y medium and grown to the mid-log phase (OD_595_ of 0.3) at 37 °C. Cells were harvested by centrifugation (6000 × *g*, 10 min), washed with phosphate-buffered saline (PBS) and then stored at −70 °C in C + Y medium with 20% glycerol^32^. Before injection, bacteria were suspended in sterile PBS containing 1% (w/v) amaranth to facilitate visualisation of successful hindbrain injection.

### Growth curves

For growth curves, bacteria were collected after an overnight culture on COS plates in fresh C + Y medium and grown until the stationary phase at 37 °C^31,33^. Next, cultures were diluted to an OD_595_ of 0.05 in the fresh C + Y medium and growth curves were performed in a plate reader (Biotek Synergy H1) for 16 h at 37 °C. For growth curves on specific sugars, C + Y medium without glucose or sucrose was used, and the appropriate sugars were added (at 1% w/v). For growth curves in oxidative stress conditions, hydrogen peroxide 30% (Supelco, Cat# 95321) was added to C + Y medium in the appropriate concentration. For growth experiments in human cerebrospinal fluid (CSF), CSF was obtained from the ventricular drain of a patient with normal pressure hydrocephalus. The CSF was collected and frozen at −80 °C until use. Bacteria were first grown until the stationary phase as described above and subsequently diluted to an OD_595_ of 0.05 in CSF.

### Transformation

Bacterial stocks for transformation were prepared by growing bacteria to an OD_595_ of 0.100 in C + Y medium at 37 °C, after which they were harvested and frozen as described above. Stocks were incubated with competence-stimulating peptide 1 (Anaspec, Cat# AS-63779) at 37 °C for 15 min before incubation with DNA constructs at 30 °C for 20 min. Next, cells were diluted 10 times in C + Y medium and incubated for 2 h. Transformants were selected by plating the suspension on Columbia blood agar plates containing erythromycin (ery) or kanamycin (kan) for knockout strains, spectinomycin (spec) for complementation strains and chloramphenicol (cam) for fluorescence and luciferase reporter strains. Antibiotic concentrations for selection used for *S. pneumoniae* were ery 0.25 µg/mL, kan 350 µg/mL, spec 100 µg/mL and cam 4.5 µg/mL. Successful transformation was confirmed by colony PCR for the knockout strains and Sanger Sequencing for the complementation strains.

### PCR for cloning

Primers were designed using Benchling Biology Software. PCR reactions were performed using 1× Phusion Hot Start Polymerase and 1× Phusion HF buffer (Thermo Scientific, Cat# F549S), supplemented with forward and reverse primers (0.4 µM each), deoxyribonucleoside triphosphates (0.2 mM), and template DNA in sterile water to the desired reaction volume. Thermal cycling consisted of an annealing step at 60 °C and an extension time of 1 min per kilobase of target sequence. DNA fragments were analysed by agarose gel electrophoresis. When necessary, bands of interest were excised from the gel and purified using a PCR purification kit (Thermo Scientific, Cat# K0691).

### Mutant generation

*S. pneumoniae* mutants were generated by chromosomal integration through double homologous recombination using linear DNA constructs flanked by approximately 1 kb homology arms upstream and downstream of the target locus. The linear constructs were assembled by Golden Gate cloning. DNA fragments were incubated in a thermocycler in the presence of the appropriate restriction enzymes, T4 DNA ligase, and 1× T4 DNA ligase buffer (New England Biolabs, Cat# B0202S), with sterile water added to reach the final reaction volume. The resulting assembly products were subsequently transformed directly into competent recipient strains.

### Construction of knockout strains

To construct the knockout mutants, the appropriate gene, including the ribosome binding site, was replaced with an erythromycin (ery^r^) or kanamycin (kan^r^) resistance marker. The upstream and downstream coding regions of the appropriate gene were amplified from genomic *S. pneumoniae* D39V. The DNA sequence containing the coding sequence and ribosome binding site of the erythromycin resistance marker was amplified together with the constitutive promoter p32 from genomic DNA of the D39V *hexA::*ery^r^ strain (Veening lab collection), while the kanamycin resistance marker was amplified together with the constitutive promoter Plac from genomic DNA of the D39V *comCDE*:*:kan*^*r*^ strain (Veening lab collection). The resistance marker and upstream and downstream fragments were assembled using restriction and ligation cloning with the appropriate restriction enzymes (i.e., XbaI, BamHI, HindIII, SpeI, or BsaI). Subsequently, the DNA product was transformed into *S. pneumoniae* D39V.

### Construction of complementation strains

To construct the *lacR* and *trxB* complementation strain, an ectopic copy of the appropriate gene under its native promoter was inserted at the ZIP locus of *S. pneumoniae* D39V. To construct this, the upstream region of the ZIP locus, along with a spectinomycin resistance cassette and the downstream region, were PCR-amplified from genomic *S. pneumoniae* D39V DNA and the D39V *cbpD::spec*^*r*^ strain (Veening lab collection). Both fragments were subsequently digested with BsmBI. Separately, the promoter and coding sequence of *lacR* and *trxB* were amplified from the genomic DNA of *S. pneumoniae* D39V and the product was digested with BsmBI. The three resulting fragments were then assembled by ligation and introduced into *S. pneumoniae* via transformation.

### Construction of fluorescently labelled strains

To construct fluorescently labelled *S. pneumoniae* strains for in vivo time-lapse imaging, the Δ*lacR* and Δ*trxB* knockout strains were transformed with a PCR product containing the *hlpA-hlpA-mScarlet-I* fragment and a downstream chloramphenicol resistance cassette. The construct was amplified from chromosomal DNA of strain *S. pneumoniae* D39V *hlpA::hlpA_hlpA-mScarlet-I-, cam*^*r*^ (VL1780)^34^. Transformants were selected on Columbia blood agar containing 4.5 µg/mL chloramphenicol and screened for red fluorescence.

### Construction of luciferase fusion strains

To construct the *lacA* reporter strains in the *S. pneumoniae* D39V wildtype and ∆*SPV_1044* knockout strain, the native promoter of *lacA* was fused to firefly luciferase and inserted at the ectopic CEP locus. To construct this, the upstream region of the CEP locus, 300 bp upstream of *lacA* to ensure the inclusion of the native promoter, was amplified from genomic *S. pneumoniae* D39V DNA, and *luc* and the downstream region from the D39V *prs1*::*lacI*-TetR, *cep*::P*htrA*-luc-Cm strain (Veening lab collection). The fragments were digested with Esp3I and assembled by ligation, then introduced into *S. pneumoniae* via transformation.

### Microtiter plate-based growth assays for luciferase tests

For *S. pneumoniae* growth assays, cells were precultured in C + Y medium until OD_595_ = 0.1 and then diluted 100-fold into 96-well microtiter plates containing fresh C + Y supplemented with appropriate concentrations of antibiotic or inducer as per each experiment. OD_595_ was measured every 10 min at 37 °C for 20 h without agitation using a TECAN Infinite F200 plate reader. In the case of luminescence assays, luciferin was added to the media at a concentration of 0.1 mg/mL to monitor gene activation by means of luciferase activity. Luminescence (relative luminescence units [RLU]) and optical density (OD595 nm) were measured every 10 min. Each growth assay was performed in biological and technical triplicate. For growth curves, the average OD_595_ value from triplicate samples was plotted. Growth curves were plotted with BactEXTRACT using default parameters^35^.

### Zebrafish husbandry and embryo care

Adult wild-type Tüpfel long fin (TL) zebrafish (*Danio rerio*) were maintained at 26 °C in aerated tanks with a 10/14 h dark/light cycle at Amsterdam UMC, while adult transparent *casper* and *Tg(mpx:*GFP*)*^*i114*^ *casper* zebrafish, expressing green fluorescently labelled neutrophils, were maintained at 28 °C in aerated 3.6-L tanks with a 10/14 h dark/light cycle at the University of Lausanne^36,37^. Zebrafish embryos were collected within the first hours post fertilisation (hpf) and kept at 28 °C in E3 medium (5.0 mM NaCl, 0.17 mM KCl, 0.33 mM CaCl·2H_2_O, 0.33 mM MgCl_2_·7H2O) supplemented with 0.3 mg/L methylene blue. At 1 day post-fertilisation (dpf), zebrafish embryos were mechanically dechorionated. All viable embryos from each clutch were used for experimentation. Sex was not determined because zebrafish embryos at this developmental stage cannot be reliably sexed. Zebrafish were used until 5 dpf.

### Zebrafish infection experiments

For survival experiments, zebrafish were infected at 2 dpf by microinjection of 1 nL of wild-type or knockout mutant *S. pneumoniae* in the hindbrain vehicle, as described previously^12^. The number of colony-forming units (CFU) was determined by quantitative plating of the injection volume before injection. Specifically, five injections were expelled into 500 µL PBS, plated, and averaged to determine the CFU per injection. After infection, zebrafish were kept in 6-well plates containing egg water (60 mg/mL sea salts (Sigma-Aldrich; S9883) in demi water) at 28 °C with 20 embryos in each experimental group. Zebrafish were monitored for survival at fixed time points until 72 h post-infection (hpi). No humane endpoints were used in this study. Experiments were performed in triplicate. Survival curves were generated with GraphPad Prism 8.0 and analysed with the log-rank (Mantel-Cox) test. Results were considered significant at *P* < 0.05. To assess the effect of reactive oxygen species inhibition in the host, infected zebrafish embryos were treated orally with 10 µM or 100 µM apocynin (Sigma-Aldrich; 178385) in 1% DMSO or 1% DMSO alone (vehicle control) by adding the compound to egg water for the whole infection course. Detailed inoculum sizes for all infection experiments are provided in Supplementary Table 2.

### Competitive index of wildtype and knockout strains in zebrafish embryos

To determine the competitive index, zebrafish embryos were injected at 2 dpf with a suspension of the knockout mutant strain and wild-type strain in a 1:1 ratio in the hindbrain ventricle. The CFUs per injection were determined by serial quantitative plating of the injection volume equivalent on COS plates. The CFU of the knockout strain (containing an erythromycin resistance marker) was determined by growth on an ery plate, while the wild-type CFU was calculated by subtracting CFU on ery COS plates from those on COS plates without antibiotics. At 24 hpi, individual zebrafish embryos were homogenised with zirconium balls in the MagNA Lyser instrument (Roche). CFUs were determined by serial quantitative plating of homogenates on COS plates containing COBA supplement (colistin sulphate 10 μg/ml and oxolinic acid 5 μg/ml; Oxoid, Cat# SR0126), which inhibits the growth of gram-negative commensals, and with or without erythromycin. CFUs of wild-type and knockout strains were determined as described above. The CFU ratio of mutant to wild-type strain was calculated before and 24 h after injection; subsequently, competitive indexes were calculated by dividing the ratio at 24 h by the ratio before injection (see formula below). A competitive index less than 1 indicates that the wild-type strain outcompetes the knockout strain.

$${{\mathrm{Competitive}}}\,{{\mathrm{index}}}=\,\frac{\frac{{{{\mathrm{CFU}}}}_{{{\mathrm{KO}}}},\,{{\mathrm{output}}}}{{{{\mathrm{CFU}}}}_{{{\mathrm{WT}}}},\,{{\mathrm{output}}}}}{\frac{{{{\mathrm{CFU}}}}_{{{\mathrm{KO}}}},\,{{\mathrm{input}}}}{{{{\mathrm{CFU}}}}_{{{\mathrm{WT}}}},\,{{\mathrm{input}}}}}$$

Experiments were performed in triplicate with 10 zebrafish per group. Plots were generated with GraphPad Prism 8.0 and analysed with a one-sample t-test. Results were considered significant at *P* < 0.05.

### Bacterial load experiments

To determine the bacterial loads, zebrafish were injected with wild-type or knockout mutant *S. pneumoniae* strains into the hindbrain ventricle as described above. At six or 24 h post-injection, zebrafish were homogenised as described above, and CFUs were determined by quantitative plating.

### RT-qPCR experiments

To isolate total RNA, zebrafish embryos (20 per biological replicate, 4 biological replicates per strain) were collected in 1.5 mL Eppendorf tubes. Excess egg water was removed, after which 350 μL of lysis buffer was added (NucleoSpin; MACHEREY-NAGEL, Cat# 740955.50). Embryos were homogenised by repeatedly passing them through a 26-gauge needle using a syringe. Total RNA was subsequently extracted using the NucleoSpin RNA isolation kit (MACHEREY-NAGEL, Cat# 740955.50). cDNA was synthesised from the isolated RNA using the iScript™ cDNA Synthesis Kit (Bio-Rad, Cat# 1708891). Primers were designed to span exon–exon boundaries (Table S4). Amplification efficiency was calculated based on the primers’ ability to double the amount of product per cycle. The qPCR reaction mixture consisted of forward and reverse primers (Table S4), cDNA, and SsoAdvanced Universal SYBR Green Supermix (Bio-Rad, Cat# 1725270). All reactions were performed in triplicate. RT-qPCR was carried out on a CFX96 Touch Real-Time PCR Detection System (Bio-Rad). Mobk13 (*mob4*) and beta-actin (*actb2*) were used as reference genes, and their average expression was used to calculate the relative gene expression using the ΔΔCt method.

### Time-lapse fluorescence imaging of zebrafish embryos

Time-lapse imaging of *Tg(mpx:*GFP*)* zebrafish embryos was performed using a Nikon AXR confocal microscope. Embryos were injected at 3 dpf, a stage characterised by increased neutrophil abundance and functional maturation relative to 2 dpf, enabling more consistent recruitment dynamics. A total of approximately 1000 CFU of red fluorescently (mScarlet-I) labelled *S. pneumoniae* D39V, ∆*lacR*, or ∆*trxB* was injected into the hindbrain ventricle. This inoculum was increased compared to the approximately 300 CFU typically used for survival experiments to allow clear visualisation of fluorescent bacteria within the hindbrain ventricle and to enable quantification of neutrophil recruitment dynamics throughout the 8-hour imaging period while maintaining embryo viability. Imaging was initiated approximately 1 h post-injection, and z-stacks were acquired at 20-min intervals for a total duration of 8 h. Following injection, anaesthetised embryos were embedded in 0.5% low melting point agarose prepared in egg water containing 60 mg/mL sea salts (Sigma-Aldrich, S9883) and supplemented with 0.02% (w/v) tricaine. Embryos were mounted in open, uncoated 8-well chamber slides and maintained at 28 °C in a temperature-controlled imaging chamber (Okolab). NIS Elements and ImageJ software were used for image processing and neutrophil quantification.

### Evaluation of pneumococcal dissemination to the central nervous system following systemic infection

To assess the potential of the *S. pneumoniae* ∆*lacR* and ∆*trxB* knock-out strains to disseminate to the central nervous system relative to the wild type, bacterial load was quantified in the head region of 2 dpf *casper* zebrafish embryos following systemic infection. Embryos were infected by microinjection of approximately 300 CFU (range 250–350) into the bloodstream via the caudal vein. At 24 hpi, embryos were anesthetized in 0.02% tricaine (Sigma-Aldrich, Cat# A5040) in egg water. Under a Nikon SMZ25 stereomicroscope, embryos were decapitated using sterile surgical blades, and each head was transferred individually to a 1.5 mL screw-cap tube containing 1.0 mm glass beads (Sigma-Aldrich, Cat# Z250473), filling approximately 25% of the tube volume. Heads were homogenised for 30 s in a FastPrep®-24 5 G bead beating system (MP Biomedicals, 11600550) to ensure complete tissue disruption. Serial ten-fold dilutions were prepared in sterile phosphate-buffered saline and plated on Columbia Blood Agar supplemented with 2% defibrinated sheep blood, 10 mg/L colistin sulfate, and 5 mg/L oxolinic acid (COBA; Oxoid, SR0126) to suppress commensal bacterial growth. Plates were incubated overnight at 37 °C, and CFUs were counted the following day and expressed as CFU per head.

### Capsule measurement by Quellung test

Appropriate strains were grown overnight to the stationary phase on chemically defined medium with either glucose and sucrose or galactose (1% w/v). Capsule visualisation was performed using the Quellung reaction by adding 3 µl of pneumococcal antisera to 3 µl of bacterial suspension on glass slides^38^. Antiserum pools A (containing antiserum against serotypes 1, 2, 4 and 5) and T (containing antiserum against serotypes 2 and 20) were used to detect the capsule, whereas antiserum pool B (containing antiserum against serotypes 3 and 8) served as a negative control (SSI Diagnostica Group). Images were acquired by phase-contrast microscopy (Leica DM6).

### Ethics approval and consent to participate

We have complied with all relevant ethical regulations for animal use. All procedures involving zebrafish in the Netherlands were performed according to local animal welfare regulations (Committee for Animal Welfare of the Amsterdam UMC - Location AMC, University of Amsterdam; Wet op de Dierproeven BWBR0003081). All procedures related to zebrafish studies at UNIL comply with the Swiss regulations on animal experimentation (cantonVaud, license number VD-H28; Animal Welfare Act SR 455 and Animal Welfare Ordinance SR 455.1). This study did not require ethical approval by a regulatory board under local law.

### Statistics and reproducibility

Data analysis was performed using Prism (Graphpad). Data shown are represented as mean of at least three biological replicates ± SEM. No statistical method was used to predetermine sample size. No zebrafish were excluded from analysis. The experiments were not randomised. The investigators were not blinded to allocation during experiments and outcome assessment. No protocol was registered before the study.

### Reporting summary

Further information on research design is available in the Nature Portfolio Reporting Summary linked to this article.

## Supplementary information

Supplementary Material

Description of Additional Supplementary Files

Data Set 1

Reporting summary
