# Supplementary material for: LacR and TrxB are key virulence factors involved in pneumococcal meningitis as identified by a genome-wide association study
Source: Commun Biol. 2026 Jul 8;9:1438. doi: 10.1038/s42003-026-10638-6 (PMC13724663; doi:10.1038/s42003-026-10638-6)
Supplement: Supplementary file 1 [file 42003_2026_10638_MOESM1_ESM.pdf]

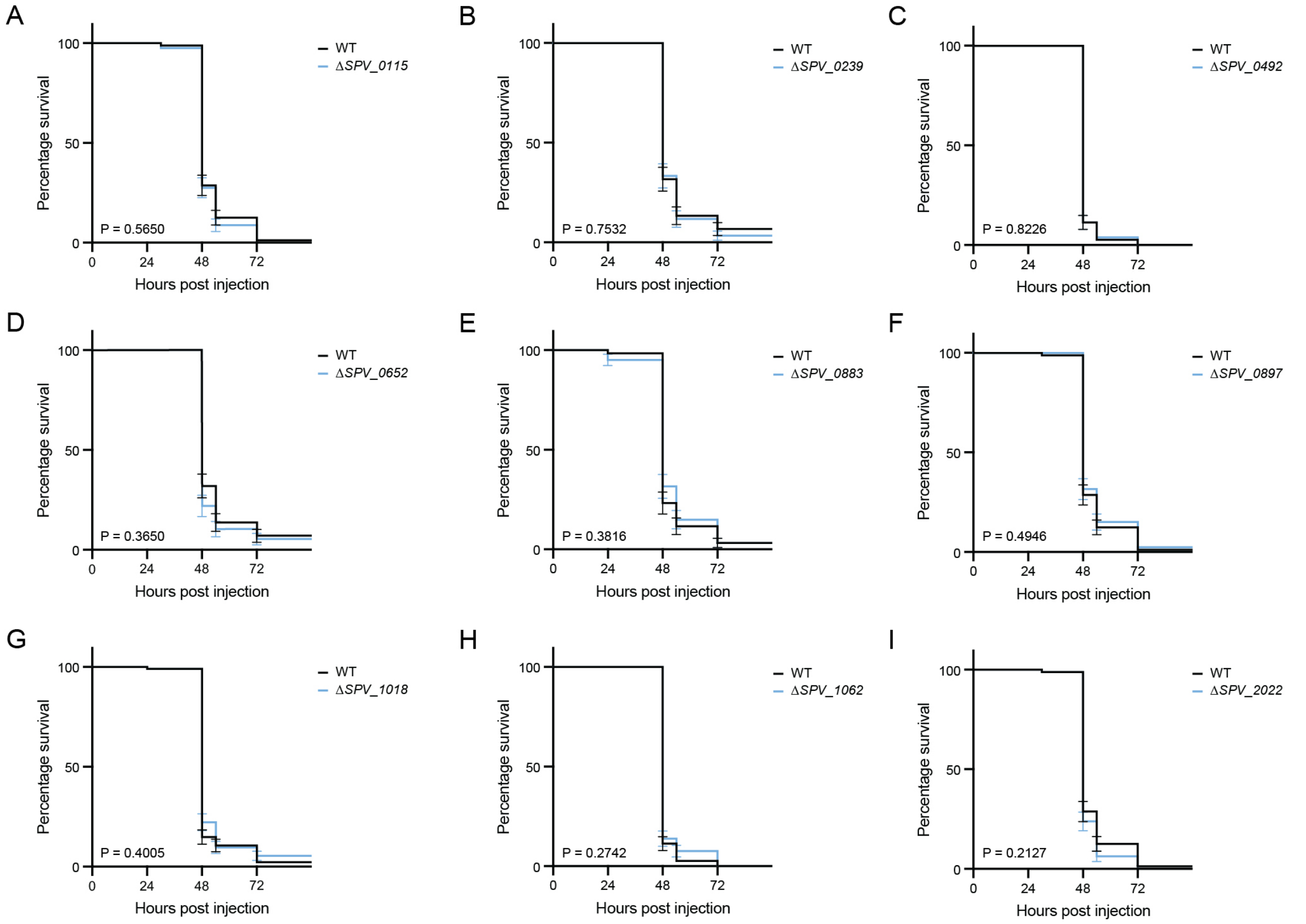

Supplementary Fig 1. Survival of zebrafish infected with mutant strains showing no attenuation of virulence compared to wild-type. (A-I) Zebrafish embryos were infected in the hindbrain with ~300 CFU wild-type (black) or knockout (blue) strains and followed for survival for 72 hours. The data represent the mean of three biological replicates with 20 larvae per group (n = 60 in total/group); P values were determined by log-rank test.

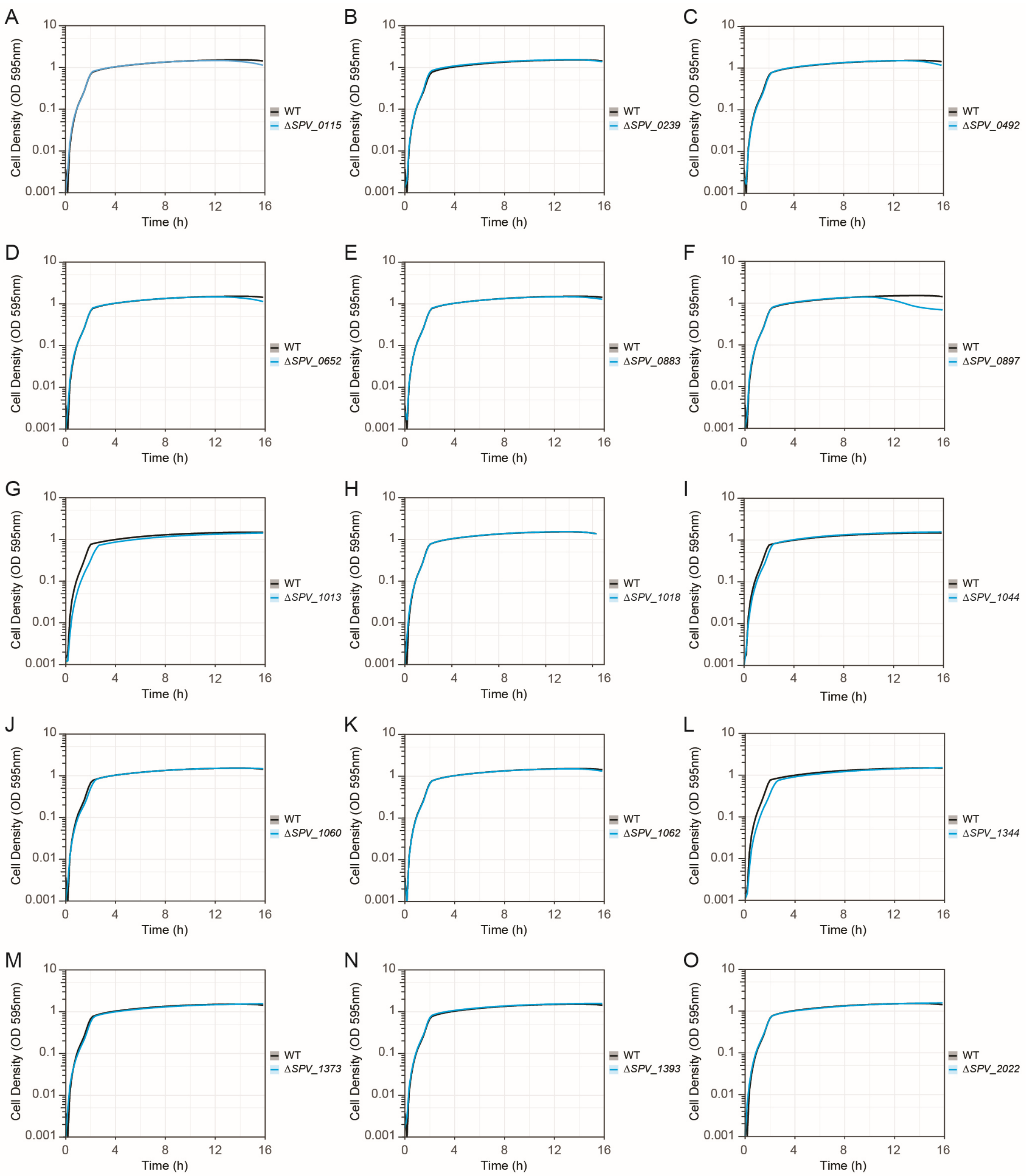

Supplementary Fig. 2. Growth curves of knockout mutant strains compared to the wild-type strain. (A-O) Growth curves of *Streptococcus pneumoniae* D39V wild-type (WT) or knockout strains in chemically defined medium. Strains were grown under approximate microaerophilic conditions in a microplate reader and the OD595 was measured every 10 minutes for 16 hours. Compared to the wild-type strain, the knockout mutant strains ΔSPV_1013 and ΔSPV_1044 show delayed growth.

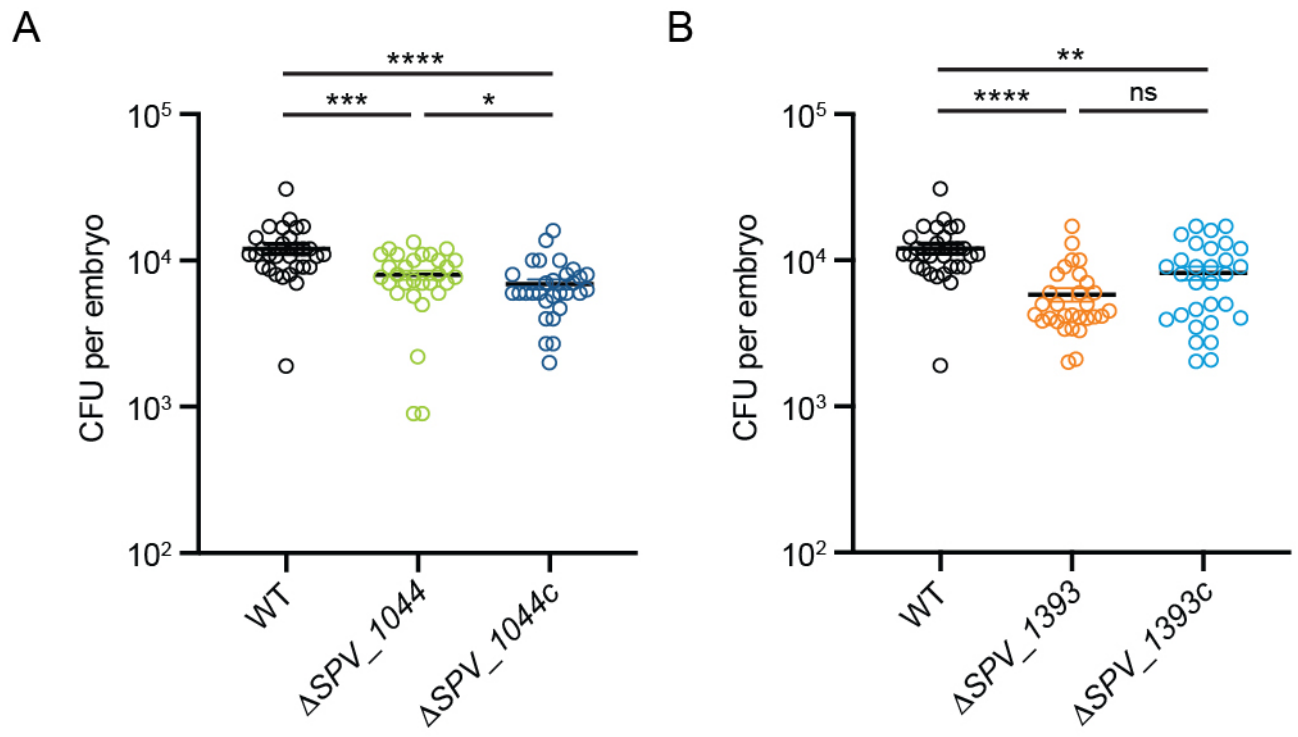

Supplementary Fig 3. Zebrafish bacterial loads at 6 hours after infection. (A-B) Bacterial load in whole zebrafish embryos infected with the indicated strains. Data are from three biological replicates with 10 embryos per group per experiment (30 per group). Each circle represents an individual zebrafish; the bar indicates the mean. Statistical analysis was performed using the Kruskal-Wallis test, * = P < 0.05, ** = P = 0.0040, **** = P < 0.0001.

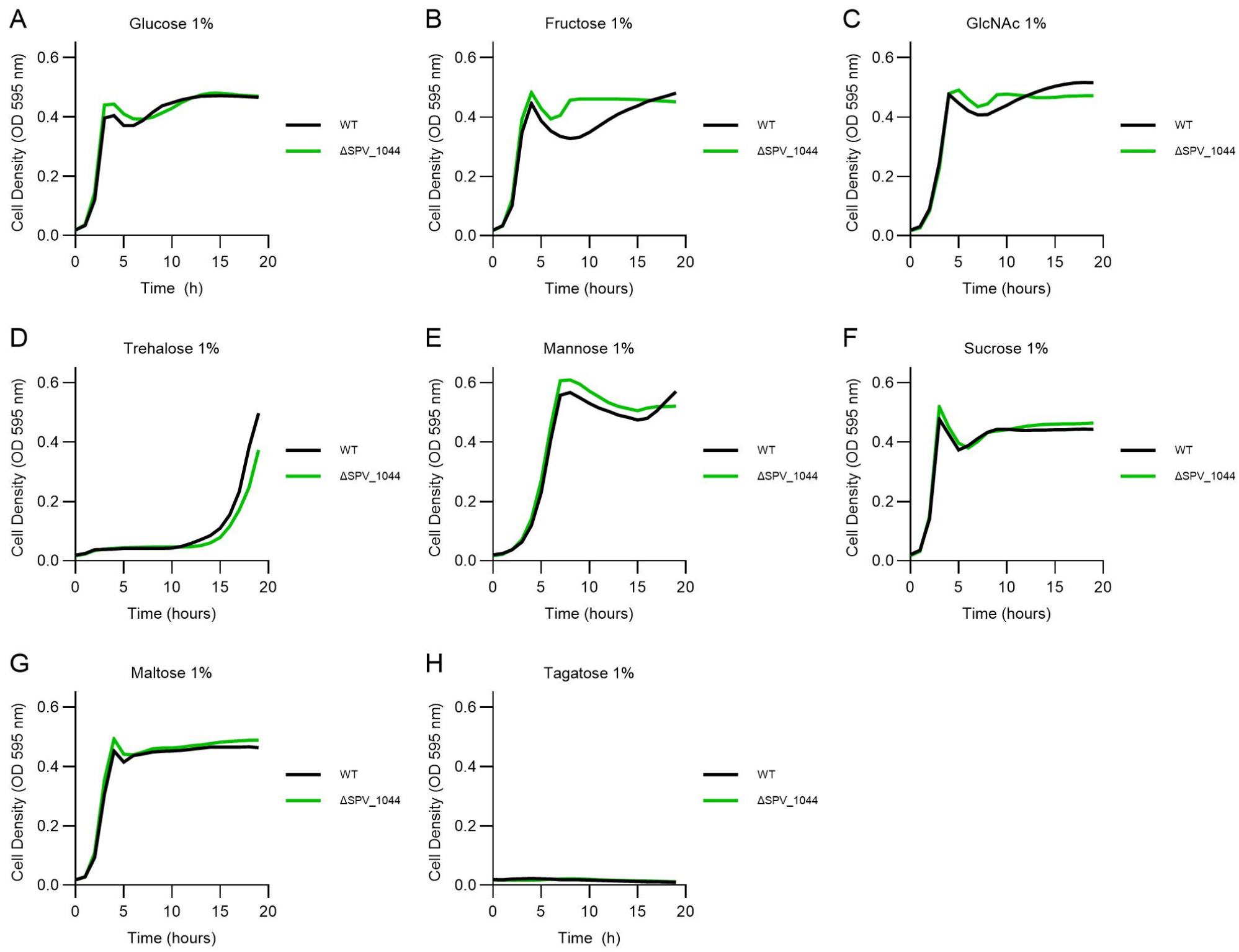

Supplementary Fig. 4. Growth curves of the *lacR* mutant in various sugars. (A-H) *Streptococcus pneumoniae* D39V wild-type (WT, black line) and SPV_1044 knockout (ΔSPV_1044, green line) strains were grown in chemically defined medium, in which sugars are omitted, supplemented with various sugars (1% w/v). In general, the growth of the knockout strain is comparable to the wild-type strain, although slight differences are seen in the growth curves on fructose and trehalose.

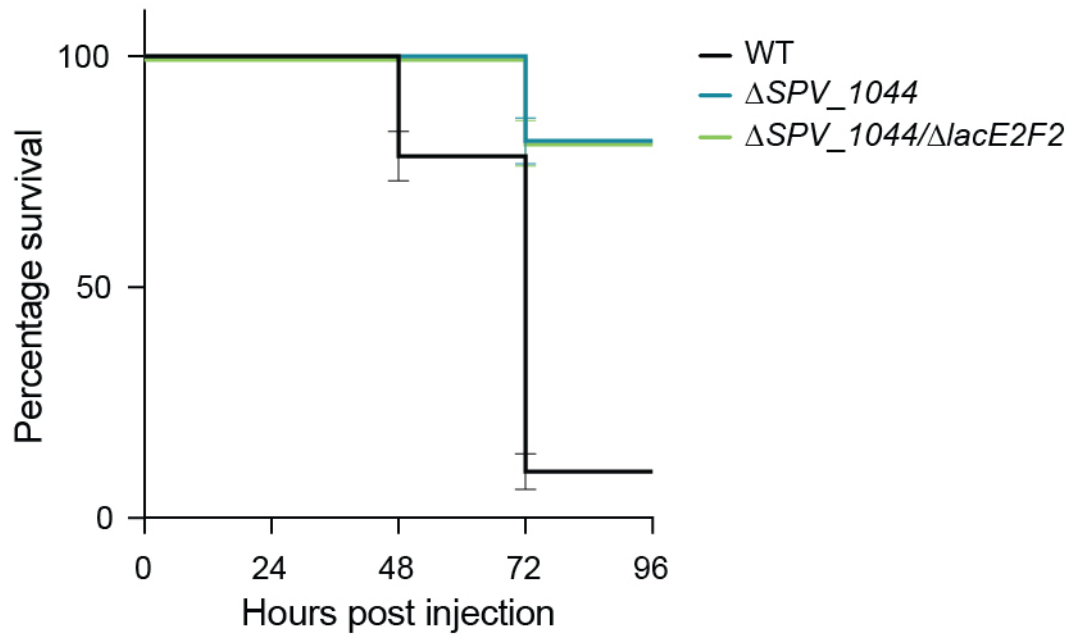

Supplementary Fig. 5. Survival curves of zebrafish infected with double ΔlacR and ΔlacE2F2 mutant compared to wild-type. Survival curves of zebrafish embryos infected in the hindbrain ventricle with ~300 colony forming units (CFU) (range 270-340) wild-type (black), ΔSPV_1044 (blue) or ΔSPV_1044 /ΔlacEF (green) strains. Zebrafish were infected by hindbrain ventricle injection and followed for survival for 72 hours. The data represent the mean ± SEM of three biological replicates with 20 embryos per group (60 in total/group); P values were determined by log-rank test.

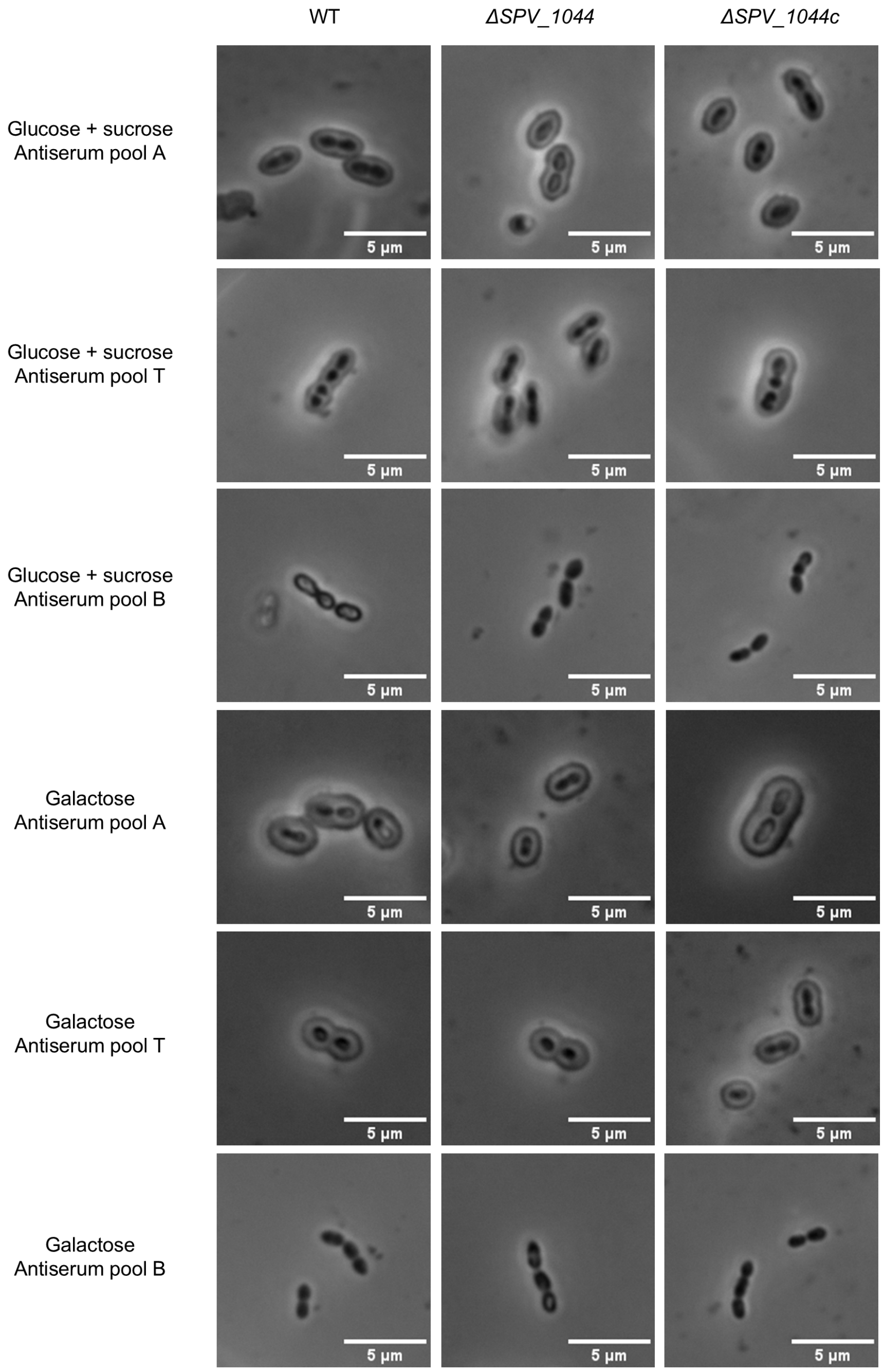

Supplementary Fig. 6. Capsule morphology is not altered in *lacR* knockout strains. *Streptococcus pneumoniae* D39V wild-type (WT), SPV_1044 knockout (ΔSPV_1044), and complemented (ΔSPV_1044c) strains were cultured in chemically defined medium supplemented with glucose and sucrose or galactose as carbon sources (1% w/v). Capsule visualization was performed using the Quellung reaction by adding pneumococcal antisera to bacterial suspensions. Antiserum pools A and T (both containing antibodies against serotype 2) were used to detect the capsule, whereas antiserum pool B (lacking serotype 2–specific antibodies) served as a negative control. Images were acquired by phase-contrast microscopy.

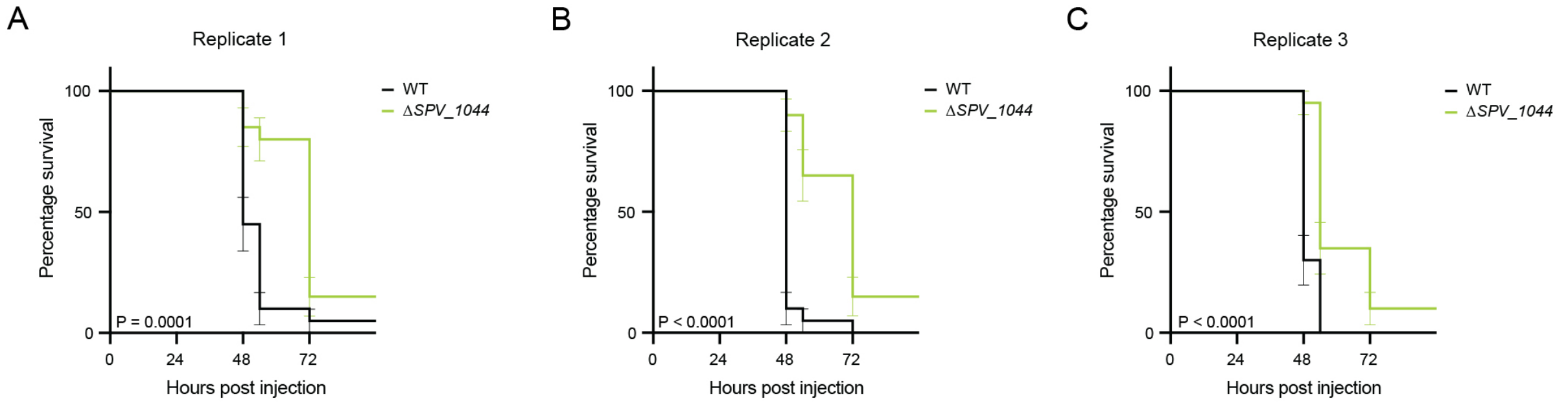

Supplementary Fig. 7. Zebrafish survival curves from independent experiments. (A-C) Survival curves of zebrafish embryos infected in the hindbrain ventricle with ~300 colony forming units (CFU) (range 130-330) wild-type (black) or ΔSPV_1044 (green) strains. Zebrafish were infected by hindbrain ventricle injection and followed for survival for 72 hours. Survival curves are shown for experiments performed on different days and did not differ significantly between experiments (determined using the log-rank test). Data represent 20 larvae per group. Statistical significance was assessed using the log-rank test.

Supplementary Table 1. Pneumococcal genetic hits from genome-wide association study associated with unfavourable outcome and mortality in patients with pneumococcal meningitis

Name	Associated with	P value	-Log (P value)	start associated region (bp)	end associated region (bp)	Genetic hits mapped to gene(s)	Gene(s) knocked out
SPV_1180	Unfavourable outcome	3.691E-05	4.432855955	1213141	1213728	SPV_1180	Not done (transformation unsuccessful)
SPV_1666	Mortality	4.072E-05	4.390192231	1679099	1679881	SPV_1666, SPV_2404	Not done (transposase)
SPV_1018	Unfavourable outcome	4.406E-05	4.355955507	921236	927127	SPV_1018	SPV_1018
SPV_0048	Mortality	4.438E-05	4.352812702	40982	41500	SPV_0048, SPV_2085	Not done (transposase)
SPV_2271	Mortality	4.438E-05	4.352812702	965072	965590	SPV_2271	Not done (transposase)
SPV_1708	Mortality	5.444E-05	4.264081883	1709063	1710292	SPV_1708	Not done (transposase)
SPV_0600	Unfavourable outcome	5.975E-05	4.22366209	625783	626973	SPV_0600	Not done (essential gene)
SPV_0761	Unfavourable outcome	6.688E-05	4.174703736	772763	772957	SPV_0761	Not done (essential gene)
SPV_1250	Unfavourable outcome	6.749E-05	4.170760572	1279399	1280223	SPV_1250	Not done (essential gene)
SPV_2129	Unfavourable outcome	9.768E-05	4.010194349	231544	232612	SPV_0240, SPV_2129, SPV_2130	Not done (transformation unsuccessful)
SPV_1373	Unfavourable outcome	1.311E-04	3.882397308	1385474	1386661	SPV_1372, SPV_1373	SPV_1372, SPV_1373
SPV_1060	Unfavourable outcome	1.338E-04	3.873543887	1085358	1087181	SPV_1060	SPV_1060
SPV_0897	Unfavourable outcome	1.386E-04	3.85823677	1052815	1053807	SPV_0896, SPV_0897, SPV_0898	SPV_0896, SPV_0897, SPV_0898
SPV_0883	Unfavourable outcome	1.437E-04	3.842543232	894813	895615	SPV_0883, SPV_0884, SPV_2263	SPV_0883, SPV_0884, SPV_2263
SPV_2143	Unfavourable outcome	1.437E-04	3.842543232	313112	313621	SPV_2143	Not done (transposase)
SPV_0238	Unfavourable outcome	1.536E-04	3.813608784	227270	230227	SPV_0238	Not done (essential gene)
SPV_2327	Unfavourable outcome	1.541E-04	3.812197361	1213821	1213964	SPV_2327	Not done (transformation unsuccessful)
SPV_0115	Mortality	1.553E-04	3.808828544	116686	118083	SPV_0114, SPV_0115	SPV_0114, SPV_0115
SPV_0652	Unfavourable outcome	1.646E-04	3.783570169	670443	671603	SPV_0652	SPV_0652
SPV_0239	Unfavourable outcome	1.820E-04	3.739928612	230421	231116	SPV_0239	SPV_0239
SPV_1393	Unfavourable outcome	1.850E-04	3.732828272	1414602	1415570	SPV_1393	SPV_1393
SPV_1344	Unfavourable outcome	1.949E-04	3.710188161	1358327	1358734	SPV_1344	SPV_1344
SPV_2022	Unfavourable outcome	1.963E-04	3.7070797	2000918	2003350	SPV_2022	SPV_2022, SPV_2023
SPV_0492	Unfavourable outcome	2.247E-04	3.648396928	498155	498712	SPV_0491, SPV_0492	SPV_0491, SPV_0492
SPV_1013	Mortality	2.272E-04	3.643591673	936481	937462	SPV_1012, SPV_1013, SPV_2268	SPV_1013, SPV_2268
SPV_1664	Unfavourable outcome	2.299E-04	3.638461029	1676081	1678048	SPV_1664	Not done (transformation unsuccessful)
SPV_0063	Unfavourable outcome	2.378E-04	3.62378815	60528	64466	SPV_0063	Not done (transformation unsuccessful)
SPV_1062	Unfavourable outcome	2.504E-04	3.601365675	1087990	1089657	SPV_1062	SPV_1061, SPV_1062, SPV_1063
SPV_1044	Unfavourable outcome	2.539E-04	3.595337299	1073586	1074529	SPV_1043, SPV_1044	SPV_1043, SPV_1044
SPV_2288	Mortality	2.556E-04	3.592439151	1032459	1033027	intergenic region	Not done (intergenic region)

Pneumococcal genetic hits identified by a previously published genome-wide association study from our group, associated with unfavourable outcome or mortality in patients with pneumococcal meningitis. The top 30 hits are shown. Knockout mutants in the *Streptococcus pneumoniae* D39V background were successfully generated for 15 loci. Mutants were not constructed for loci mapping to intergenic regions, transposons or essential genes.

Supplementary Table 2. CFU inoculum used in zebrafish infection experiments.

Experiment	Corresponding figure(s)	CFU WT (range)	CFU knockout strain (range)	CFU complemented strain (range)
Survival experiment *S. pneumoniae* D39V WT vs Δ*SPV_0115*	Supplementary Figure 1	~310 (250-350)	~280 (240-330)	N/A
Survival experiment *S. pneumoniae* D39V WT vs Δ*SPV_0239*	Supplementary Figure 1	~310 (260-390)	~290 (220-350)	N/A
Survival experiment *S. pneumoniae* D39V WT vs Δ*SPV_0492*	Supplementary Figure 1	~290 (260-330)	~300 (280-330)	N/A
Survival experiment *S. pneumoniae* D39V WT vs Δ*SPV_0652*	Supplementary Figure 1	~310 (260-390)	~280 (240-330)	N/A
Survival experiment *S. pneumoniae* D39V WT vs Δ*SPV_0883*	Supplementary Figure 1	~290 (250-310)	~250 (230-270)	N/A
Survival experiment *S. pneumoniae* D39V WT vs Δ*SPV_0897*	Supplementary Figure 1	~310 (250-350)	~330 (280-400)	N/A
Survival experiment *S. pneumoniae* D39V WT vs Δ*SPV_1013*	Figure 2B	~310 (250-360)	~300 (280-310)	N/A
Survival experiment *S. pneumoniae* D39V WT vs Δ*SPV_1018*	Supplementary Figure 1	~270 (210-320)	~330 (310-360)	N/A
Survival experiment *S. pneumoniae* D39V WT vs Δ*SPV_1044*	Figure 2B	~320 (280-350)	~340 (300-380)	N/A
Survival experiment *S. pneumoniae* D39V WT vs Δ*SPV_1060*	Figure 2B	~290 (250-310)	~340 (300-400)	N/A
Survival experiment *S. pneumoniae* D39V WT vs Δ*SPV_1062*	Supplementary Figure 1	~290 (260-330)	~310 (280-330)	N/A
Survival experiment *S. pneumoniae* D39V WT vs Δ*SPV_1344*	Figure 2B	~320 (280-350)	~260 (230-280)	N/A
Survival experiment *S. pneumoniae* D39V WT vs Δ*SPV_1373*	Figure 2B	~290 (250-310)	~350 (330-380)	N/A
Survival experiment *S. pneumoniae* D39V WT vs Δ*SPV_1393*	Figure 2B	~310 (260-390)	~310 (290-330)	N/A
Survival experiment *S. pneumoniae* D39V WT vs Δ*SPV_2022*	Supplementary Figure 1	~310 (250-350)	~330 (320-380)	N/A
Competitive index *S. pneumoniae* D39V WT vs Δ*SPV_1013*	Figure 2C	~210 (170-250)	~190 (160-210)	N/A
Competitive index *S. pneumoniae* D39V WT vs Δ*SPV_1044*	Figure 2C, Supplementary Figure 7	~240 (160-330)	~190 (130-240)	N/A
Competitive index *S. pneumoniae* D39V WT vs Δ*SPV_1060*	Figure 2C	~110 (100-120)	~140 (90-160)	N/A
Competitive index *S. pneumoniae* D39V WT vs Δ*SPV_1344*	Figure 2C	~300 (290-300)	~250 (200-300)	N/A
Competitive index *S. pneumoniae* D39V WT vs Δ*SPV_1373*	Figure 2C	~290 (230-330)	~260 (240-300)	N/A
Competitive index *S. pneumoniae* D39V WT vs Δ*SPV_1393*	Figure 2C	~140 (130-180)	~160 (100-190)	N/A
Survival experiment *S. pneumoniae* D39V WT vs Δ*SPV_1044* vs Δ*SPV_1044c*	Figure 3B	~260 (240-290)	~230 (190-290)	270 (250-280)
Survival experiment *S. pneumoniae* D39V WT vs Δ*SPV_1393 vs* Δ*SPV_1393c*	Figure 3B	~260 (240-290)	~290 (250-330)	210 (190-220)
Bacterial load and gene expression experiment *S. pneumoniae* D39V WT vs Δ*SPV_1044* (6 hpi)	Figure 3G-3H, Supplementary Figure 3	~280 (230-340)	~330 (240-390)	300 (210-390)
Bacterial load and gene expression experiment *S. pneumoniae* D39V WT vs Δ*SPV_1393* (6 hpi)	Figure 3G-3H, Supplementary Figure 3	~280 (230-340)	~280 (230-330)	290 (230-320)
Bacterial load and gene expression experiment *S. pneumoniae* D39V WT vs Δ*SPV_1044* (24 hpi)	Figure 3C, 3G-3H	~250 (200-310)	~250 (180-300)	280 (200-340)
Bacterial load and gene expression experiment *S. pneumoniae* D39V WT vs Δ*SPV_1393* (24 hpi)	Figure 3C, 3G-3H	~250 (200-310)	~240 (120-330)	270 (230-330)
Brain invasion *S. pneumoniae* D39V WT vs Δ*SPV_1044*	Figure 3D	~330 (300-350)	~340 (320-350)	N/A
Brain invasion *S. pneumoniae* D39V WT vs Δ*SPV_1393*	Figure 3D	~330 (300-350)	~330 (300-350)	N/A
Neutrophil recruitment experiment *S. pneumoniae* D39V WT vs Δ*SPV_1044*	Figure 3E	~1000 (950-1090)	~1000 (970-990)	N/A
Neutrophil recruitment experiment *S. pneumoniae* D39V WT vs Δ*SPV_1393*	Figure 3E	~1000 (950-1090)	~1000 (1000-1050)	N/A
Survival experiment *S. pneumoniae* D39V WT vs Δ*SPV_1044* , Δ*lacE2-F2*	Supplementary Figure 5	~300 (270-320)	~330 (320-340)	N/A

Supplementary Table 3. Strains and plasmids

Strain	Description	Reference
D39V	pathogenic, serotype 2	Slager et al. (13)
KR01	D39V Δ*SPV_0115*::ery^r	This study
KR02	D39V Δ*SPV_0239*::ery^r	This study
KR03	D39V Δ*SPV_0492*::ery^r	This study
KR04	D39V Δ*SPV_0652*::ery^r	This study
KR05	D39V Δ*SPV_0883*::ery^r	This study
KR06	D39V Δ*SPV_0897*::ery^r	This study
KR07	D39V Δ*SPV_1013*::ery^r	This study
KR08	D39V Δ*SPV_1018*::ery^r	This study
KR09	D39V Δ*SPV_1044*::ery^r	This study
KR10	D39V Δ*SPV_1060*::ery^r	This study
KR11	D39V Δ*SPV_1062*::ery^r	This study
KR12	D39V Δ*SPV_1344*::ery^r	This study
KR13	D39V Δ*SPV_1373*::ery^r	This study
KR14	D39V Δ*SPV_1393*::ery^r	This study
KR15	D39V Δ*SPV_2022*::ery^r	This study
KR16	D39V Δ*SPV_1344*::ery^r, ZIP::*SPV_1344* (spec) complemented	This study
KR17	D39V Δ*SPV_1373*::ery^r, ZIP::*SPV_1373* (spec) complemented	This study
KR18	D39V, CEP::P*lac-luc*	This study
KR19	D39V Δ*SPV_1044*::ery^r, CEP::P*lac-luc*	This study
KR20	D39V Δ*lacE2F2*::kanr	This study

Supplementary Table 4. Primers

Oligonucleotide name	Sequence (5' to 3')	Description
SPV0115_LF_FW	ATGTTAAAAAAATATCCTTGTACGATGCAACATGAC	Construction of *SPV_0115::ery*^{r}
SPV0115_LF_RV	TTTTTCTAGATTAGCCATAAGTAACCTCCTCTGTATCTAAC	Construction of *SPV_0115::ery*^{r}
SPV0115_RF_FW	TTTTGGATCCGAAAGATATCTTATCTAGGTAACTAAGTGC	Construction of *SPV_0115::ery*^{r}
SPV0115_RF_RV	AAGCGAAGGAAAGATAAAAAAGCCGAG	Construction of *SPV_0115::ery*^{r}
P32-eryR_XbaI_FW	TTTTCTAGAATTATTTCCTCCCGTTAAATAATAGATAACTATTAAAAA	Construction of knockout mutants
P32-eryR_BamHI_RV	TTTTGGATCCGGATTTTTGTGAGCTTGGACTAGAAAAAAACT	Construction of knockout mutants
SPV0239_LF_FW	ATGAGTTTTTATAATCATAAAGAAATTGAGCCTAAGTGGC	Construction of *SPV_0239::ery*^{r}
SPV0239_LF_RV	TTTTTCTAGAAGCTTTCTCCTTTTTTCACTTTTCAAACT	Construction of *SPV_0239::ery*^{r}
SPV0239_RF_FW	TTTTGGATCCGAGGTGTTGAGATGATTACTATTAAAAAG	Construction of *SPV_0239::ery*^{r}
SPV0239_RF_RV	CTTTTTATTATCCCATAGTTCACGAATTTTGTCAAAGG	Construction of *SPV_0239::ery*^{r}
SPV0652_LF_FW	ATGATTCCTGTAGTTATTGAACAAACAAGCC	Construction of *SPV_0652::ery*^{r}
SPV0652_LF_RV	TTTTTCTAGAGTTAAGAATATACCGAATTATCAGAAAATTGTCAATGT	Construction of *SPV_0652::ery*^{r}
SPV0652_RF_FW	TTTTGGATCCTTTAATACTCTTCGAAAATCTCTTCAAACTG	Construction of *SPV_0652::ery*^{r}
SPV0652_RF_RV	TTACACCTTCTCTTTCACATTCTTACCAAGG	Construction of *SPV_0652::ery*^{r}
SPV0883_LF_FW	ATGAAACCTGAAAAACCTAAAAAATTGGGTCT	Construction of *SPV_0883::ery*^{r}
SPV0883_LF_RV	TTTTTCTAGATGTGTACTATTTTTGGTTCATTTTACTATATTTTATAAGT	Construction of *SPV_0883::ery*^{r}
SPV0883_RF_FW	TTTTGGATCCAACATGATTTATGAAATGTCAGACCT	Construction of *SPV_0883::ery*^{r}
SPV0883_RF_RV	TTATTCAAAAATTCCAGCTAGAACATTTACTTGTCCT	Construction of *SPV_0883::ery*^{r}
SPV0897_LF_FW	AAAAATCCCAGTCGCTAGACTAGGG	Construction of *SPV_0897::ery*^{r}
SPV0897_LF_RV	TTTTTCTAGAAAGAAGATAAGCTAGGTAGTTCTTACCTGG	Construction of *SPV_0897::ery*^{r}
SPV0897_RF_FW	TTTTGGATCCTTACTCTTTGGGTAACTTGCC	Construction of *SPV_0897::ery*^{r}
SPV0897_RF_RV	GATAAAAACATCACAAGGAAGCAGTCATGAAAAA	Construction of *SPV_0897::ery*^{r}
SPV1018_LF_FW	TTAACTTTCTTTTTTACCTAAAACCAGTGATTTAACGG	Construction of *SPV_1018::ery*^{r}
SPV1018_LF_RV	TTTTTCTAGATATATAGGGTAAAAGATAGGCTAAAAGATAGGGTAAAAGG	Construction of *SPV_1018::ery*^{r}
SPV1018_RF_FW	TTTTGGATCCACCTTAATTATACTCTTAATTTACAAAAAAGTCTTAAAATTGAG	Construction of *SPV_1018::ery*^{r}
SPV1018_RF_RV	ATGACACTTCCAGTTAGAAAATCCCTGC	Construction of *SPV_1018::ery*^{r}
P32-eryR_HindIII_RV	TTTTAAGCTTGGATTTTTGTGAGCTTGGAC	Construction of knockout mutants
SPV1062_LF_FW	TTATTCTTCATCCATACTCAAGACGCTGAG	Construction of *SPV_1062::ery*^{r}
SPV1062_LF_RV	TTTTACTAGTTACTCCTAGCAGGGTATGGT	Construction of *SPV_1062::ery*^{r}
SPV1062_RF_FW	TTTTGGATCCTTATTCATTTTTAAATTGACTATGCGCC	Construction of *SPV_1062::ery*^{r}
SPV1062_RF_RV	CTCTGGAAGATGCGATTACTGCC	Construction of *SPV_1062::ery*^{r}
P32-eryR_SpeI_FW	TTTTACTAGTTTATTTCCTCCCGTTAAATAATAGATAACTATTAAAAATAGAC	Construction of knockout mutants
SPV2022_LF_FW	CTCTCTCCCTTTCTACTACCAGAACTCC	Construction of *SPV_2022::ery*^{r}
SPV2022_LF_RV	TTTTTCTAGAAAGAATAAAAGTATCAGCATCTGACCATAAGTC	Construction of *SPV_2022::ery*^{r}
SPV2022_RF_FW	TTTTGGATCCTTGACTATTATACCGAAATTTTCTCATTTTTAAAAGAA	Construction of *SPV_2022::ery*^{r}
SPV2022_RF_RV	ATGACAGAAATTAGACTAGAGCACGTCAG	Construction of *SPV_2022::ery*^{r}
SPV0492_LF_FW	ATGAGTTACGAACAAGAATTTATGAAGGAATTTGAAG	Construction of *SPV_0492::ery*^{r}
SPV0492_LF_RV	TTTTTCTAGAGGCTCTATTGTAGCAAGTTTTTCTCG	Construction of *SPV_0492::ery*^{r}
SPV0492_RF_FW	TTTTGGATCCGATGAGCGATGTAAAAGAGG	Construction of *SPV_0492::ery*^{r}
SPV0492_RF_RV	TTAATTACTTTTATTGACAATTTTAACCAAACCAATTTTTGAAAGTT	Construction of *SPV_0492::ery*^{r}
SPV1013_LF_FW	CTCAGCCTCGTTGGAATTTTTCATCAC	Construction of *SPV_1013::ery*^{r}
SPV1013_LF_RV	TTTTGCTAGCAAGAAGATTATACCACATTGTGTACT	Construction of *SPV_1013::ery*^{r}
SPV1013_RF_FW	TTTTGGATCCTTATTTTTTAAGGTTGTAGAATGATTTCAATCCAC	Construction of *SPV_1013::ery*^{r}
SPV1013_RF_RV	ATGTCAATTATTACTGATGTTTACGCTCGC	Construction of *SPV_1013::ery*^{r}
SPV1044_LF_FW	ATGGAAACTTACTACAAAGCCATTAACTGGAA	Construction of *SPV_1044::ery*^{r}
SPV1044_LF_RV	TTTTTCTAGATAGTCTAAACCGTAGTTATAGTCATCGTCTTG	Construction of *SPV_1044::ery*^{r}
SPV1044_RF_FW	TTTTGGATCCTAAACTGAAAGAGCCTGGG	Construction of *SPV_1044::ery*^{r}
SPV1044_RF_RV	TAGAATTAGTCATTAGATATAGAATTTTAGTGAGTCCAAAAGATGT	Construction of *SPV_1044::ery*^{r}
SPV1060_LF_FW	TTAAGCGATTGCTTCATCTATATAAGCATAAAGTTTTTCT	Construction of *SPV_1060::ery*^{r}
SPV1060_LF_RV	TTTTTCTAGATAGTTTTTATACTCTTCGAAAATCTCTTCAAACCAC	Construction of *SPV_1060::ery*^{r}
SPV1060_RF_FW	TTTTGGATCCCTATTTATTATAAATTGTTTTTGATATTTTGACAAGACCA	Construction of *SPV_1060::ery*^{r}
SPV1060_RF_RV	ATGACAGATTATTATGTAATTGGAGATGTTCACGG	Construction of *SPV_1060::ery*^{r}
SPV1344_LF_FW	TTCTGTGCCTTTCTTGTGTTTGTGG	Construction of *SPV_1344::ery*^{r}
SPV1344_LF_RV	TTTTTCTAGAATGTATAGTGCATTGAAGTTAAAATAGTACACTTTGAT	Construction of *SPV_1344::ery*^{r}
SPV1344_RF_FW	TTTTGGATCCCCTCCCCACTCCT	Construction of *SPV_1344::ery*^{r}
SPV1344_RF_RV	ACAAACTAAAATTATGTGATACTTCACATAATTTTCTTTAGAAA	Construction of *SPV_1344::ery*^{r}
SPV1373_LF_FW	TTATGGTTTGATACGGTGCAACATACG	Construction of *SPV_1373::ery*^{r}
SPV1373_LF_RV	TTTTTCTAGAAAAAGAAAAGGAAAAATAATGACAAAACGTGTAACG	Construction of *SPV_1373::ery*^{r}
SPV1373_RF_FW	TTTTGGATCCTCATAGGCCCTCCTTATTGAC	Construction of *SPV_1373::ery*^{r}
SPV1373_RF_RV	GTGACTGAAAAAGTAAGAACTGTTTCACTTTCAAA	Construction of *SPV_1373::ery*^{r}
SPV1393_LF_FW	CTATTTTTTTTCATGTTTCCATCCTCCTAGCAAT	Construction of *SPV_1393::ery*^{r}
SPV1393_LF_RV	TTTTTCTAGAAGTTTCTCCTTTGTTTTTTCTAGTCAGTTTATTTTATCA	Construction of *SPV_1393::ery*^{r}
SPV1393_RF_FW	TTTTGGATCCAAAAGAACCACGAGTCACA	Construction of *SPV_1393::ery*^{r}
SPV1393_RF_RV	ATGAGTTTCACAGTAGCAGTAAAAGAAGAAATCC	Construction of *SPV_1393::ery*^{r}
SPV0115_cPCR_FW	ACGGTTGAAAATAAATTAATTCAAGAACTACTGGG	Colony PCR *SPV_0115::eryr*
SPV0239_cPCR_FW	TCAAAATTAATGAGATACAGTATACAGTTTTTTCTTTAAACACATTTCA	Colony PCR *SPV_0239::eryr*
SPV0652_cPCR_FW	GAAAGCAGAGCAAAAATTTGACCTTTTTTGAC	Colony PCR *SPV_0652::eryr*
SPV0883_cPCR_FW	AGTCGGATCAAGCTAGGGAATTGT	Colony PCR *SPV_0883::eryr*
SPV0897_cPCR_FW	TAAATAACCTCTTTCGGAATTTTCTAAAAATTTCTATAATAGAAG	Colony PCR *SPV_0897::eryr*
SPV1018_cPCR_FW	TTCCTCGACCAACTTGGGATAGTATAGT	Colony PCR *SPV_1018::eryr*
SPV1062_cPCR_FW	TCGAAGAGTATAAAAACTAAAAAAGCAGACCATCT	Colony PCR *SPV_1062::eryr*
SPV2022_cPCR_FW	GTCCATCCTTAGCCATAATGACATGATAACC	Colony PCR *SPV_2022::eryr*
SPV0492_cPCR_FW	AGACGGAGAATTCCAAGGAGTGTT	Colony PCR *SPV_0492::eryr*
SPV1013_cPCR_FW	CAATGTAGAAGACCCGCTTGCC	Colony PCR *SPV_1013::eryr*
SPV1044_cPCR_FW	AAAGTCGGGCTTCCGACTTTTTG	Colony PCR *SPV_1044::eryr*
SPV1060_cPCR_FW	AGCACTTTTAATTTGGTGACTTGAGTTAACAC	Colony PCR *SPV_1060::eryr*

SPV1344_cPCR_FW	CAATTTCTCTATTTGATTTCCCTTCTTTTTTCCATCT	Colony PCR *SPV_1344::eryr*
SPV1373_cPCR_FW	TGAGGTTCGCTCACAATAAACTATTATACTAGAAAAAAT	Colony PCR *SPV_1373::eryr*
SPV1393_cPCR_FW	CGTTTTTACCTGCCTAGCACTGG	Colony PCR *SPV_1393::eryr*
Standard_cPCR_RV	GAAACACGCCAAAGTAAACAATTTAAGTACCA	Colony PCR knockout mutants
comp_ISU_FW	GCCAATAAATTGCTTCCTTGTTTTG	Construction of complementation mutants
C_SPV_1044_ISU_RV	AGCGTCACGTCTCCCATTATTTTTCCTCCTTATTTATTTAGA	Construction of *SPV_1044::ery^r* complemented
C_SPV_1044_FW	AGCGTCACGTCTCGAATGCTCAAACAAGAAAAATTA	Construction of *SPV_1044::ery^r* complemented
C_SPV_1044_RV	AGCGTCACGTCTCGGCTTAAGGCTTGATGACTTTAAT	Construction of *SPV_1044::ery^r* complemented
C_SPV_1044_ISD_FW	AGCGTCACGTCTCCAAGCCGGATCCCTCCAGTAAC	Construction of *SPV_1044::ery^r* complemented
comp_ISD_RV	ATGACACGGATTTTAAGAATAATTCTTTC	Construction of complementation mutants
C_SPV_1393_FW	GAGTCACGTCTCCAATGTCTCAACTCTATGATATTACCA	Construction of *SPV_1393::ery^r* complemented
C_SPV_1393_RV	GAGTCACGTCTCCGCTTATAAACTGGTAGAGTGTTTTGG	Construction of *SPV_1393::ery^r* complemented
C_SPV_1393_ISU_RV	GAGTCACGTCTCGCATTATTTTTCCTCCTTATTTATTTAGATCTTAA	Construction of *SPV_1393::ery^r* complemented
C_SPV_1393_ISD_FW	GAGTCACGTCTCGAAGCCGGATCCCTCCAGTAA	Construction of *SPV_1393::ery^r* complemented
C_cPCR_spec_FW	CTATCGGGGAGAGTGTGATGATAAG	Colony PCR of complementation mutants
C_cPCR_spec_RV	TGCGTTTTCTTGTGGAGATATTTCC	Colony PCR of complementation mutants
C_cPCR_alg_FW	GCACCACCATATAGAAGGTTAGAAAC	Colony PCR of complementation mutants
C_cPCR_1393_RV	ATCTGATGAAACTGAAACCATTGACC	Construction of *SPV_1044::ery^r* complemented
C_cPCR_1044_RV	CATATCTGAAACGGCTATTTCGTCC	Construction of *SPV_1044::ery^r* complemented
F-os_CEP	TAAAACAAACCGCATCCG	Construction of luciferase reporter strain
CEP_PlacA_upR	CCTTGACGTCTCCtaCCGTGTTGATCACCGTCT	Construction of luciferase reporter strain
placA_F	CCTTGACGTCTCGGGTAGAAGCCAATCAGGTTGAA	Construction of luciferase reporter strain
placA_R	GATGGCGTCTCGTCCTTTAATCCACAAGGTGGAAG	Construction of luciferase reporter strain
CEP_placA_downF	GATGGCGTCTCGAGGAGGAATAATGAGATCCG	Construction of luciferase reporter strain
P3-luc_R	ATTAGGCTCTTTGTCAACTG	Construction of luciferase reporter strain
tnfa_FW	GCTGGATCTTCAAAGTCGGGTGTA	Measuring zebrafish cytokine gene expression
tnfa_RV	TGTGAGTCTCAGCACACTTCCATC	Measuring zebrafish cytokine gene expression
il6_FW	CACTCAGAGACGAGCAGTTTG	Measuring zebrafish cytokine gene expression
il6_RV	TCAGGACGCTGTAGATTCGC	Measuring zebrafish cytokine gene expression
il1b_FW	CAATGCACGATTTGCGATCAG	Measuring zebrafish cytokine gene expression
il1b_RV	GACATGCTGAAGCGCACTTTC	Measuring zebrafish cytokine gene expression
cxcl8a_FW	GTCGCTGCATTGAAACAGAA	Measuring zebrafish cytokine gene expression
cxcl8a_RV	CTTAACCCATGGAGCAGAGG	Measuring zebrafish cytokine gene expression
cxcl8b_FW	CTACCGAGACGTGGGTGATT	Measuring zebrafish cytokine gene expression
cxcl8b_RV	GCTCGGTGAATGGTCATTTT	Measuring zebrafish cytokine gene expression
il10_FW	CGGGATATGGTGAAATGCAAGAG	Measuring zebrafish cytokine gene expression
il10_RV	AGCAAATCAAGCTCCCCCATAG	Measuring zebrafish cytokine gene expression
mob4_FW	CACCCGTTTCGTGATGAAGTACAA	Reference gene for measuring zebrafish cytokine gene expression
mob4_RV	GTTAAGCAGGATTTACAATGGAG	Reference gene for measuring zebrafish cytokine gene expression
actb_RV	AATTGCCGCACTGGTTGTTG	Reference gene for measuring zebrafish cytokine gene expression
actb_RV	ACCAACCATGACACCCTGATG	Reference gene for measuring zebrafish cytokine gene expression
lacE2F2_LF_FWD_BsaI	GTCAGGTCTCAAAGCGGCTAGAGACTCTGAAATAGTCTCC	Construction of *lacE2F2::kanr lacR::eryr*
lacE2F2_LF_REV_BsaI	CTAGTCTATAATCTGAGTTTCCGC	Construction of *lacE2F2::kanr lacR::eryr*
lacE2F2_RF_FWD_BsaI	CAAGTTAATACAAGGTGAGTGTTAC	Construction of *lacE2F2::kanr lacR::eryr*
lacE2F2_RF_REV_BsaI	GTCAGGTCTCAGCAGGATAGAATTCTCCTTATATAG	Construction of *lacE2F2::kanr lacR::eryr*
kanR_FWD_BsaI	GTCAGGTCTCACTGCTCGAAGATTTCAGCTTG	Construction of *lacE2F2::kanr lacR::eryr*
kanR_REV_BsaI	GTCAGGTCTCAGCTTCTAGCAAAAAACTGGACG	Construction of *lacE2F2::kanr lacR::eryr*
lacE2F2_cPCR_LF_REV	CTAATGACTAATTCTACTAGTC	Construction of *lacE2F2::kanr lacR::eryr*
lacE2F2_cPCR_RF_FWD	CTATACTTAAATCACAAGTTAATAC	Construction of *lacE2F2::kanr lacR::eryr*
hlpA_LF_FWD	AACAAGTCAGCCACCTGTAG	Construction of fluorescently labeled *S. pneumoniae* strain
hlpA_RF_REV	CGTGGCTGACGATAATGAGG	Construction of fluorescently labeled *S. pneumoniae* strain
