# Supplementary material for: LacR and TrxB are key virulence factors involved in pneumococcal meningitis as identified by a genome-wide association study
Source: Commun Biol. 2026 Jul 8;9:1438. doi: 10.1038/s42003-026-10638-6 (PMC13724663; doi:10.1038/s42003-026-10638-6)
Supplement: Supplementary file 2 [file 42003_2026_10638_MOESM2_ESM.pdf]

Description of Additional Supplementary Files

File name: Supplementary Data

Description: The source data behind the graphs in the paper
